# TCF4 promotes apoptosis and Wnt/β-catenin signaling pathway in acute kidney injury via transcriptional regulation of COX7A2L

**DOI:** 10.1371/journal.pone.0307667

**Published:** 2024-11-05

**Authors:** Minhui Xi, Jingyuan Lu, Hualin Qi

**Affiliations:** Department of Nephrology, Shanghai Pudong New Area People’s Hospital, Shanghai, China; Charotar Institute of Applied Sciences: P D Patel Institute of Applied Sciences, INDIA

## Abstract

**Background:**

Acute kidney injury (AKI) is still a serious kidney illness with high morbidity and death rates, and it’s crucial to comprehend the underlying molecular causes.

**Methods:**

Bioinformatics analysis was performed on GSE139061 and GSE30718 data sets, and COX7A2L was screened out. The role of COX7A2L in H/R-treated cells and its transcriptional regulation with TCF4 were assessed. *In vitro* experiments analyzed the regulation of COX7A2L and TCF4 on the proliferation, apoptosis, and Wnt/β-catenin signaling pathway of H/R-treated cells.

**Results:**

COX7A2L as a hub gene was downregulated in AKI samples. In H/R-treated cells, COX7A2L overexpression inhibited apoptosis and promoted cell proliferation, while COX7A2L knockdown promoted apoptosis and inhibited cell proliferation. Notably, TCF4 exhibited a significant positive correlation with COX7A2L. TCF4 overexpression-induced apoptosis was lessened and improved cell proliferation was countered by COX7A2L knockdown, according to rescue study findings. Besides, we discovered that TCF4 overexpression increased the expression of proteins linked to the Wnt/β-catenin signaling pathway (c-myc, β-catenin, and cyclin D1), while underexpression of COX7A2L counteracted this effect.

**Conclusion:**

The study revealed the pivotal role of COX7A2L in AKI, which is regulated by TCF4 and modulates the Wnt/β-catenin signaling pathway, highlighting its potential as a therapeutic target.

## Introduction

Characterized by a rapid decline in renal function that reduces urine output and impairs kidney filtration, acute kidney injury (AKI) is a perilous condition [1, 2]. Recognized globally, AKI not only demonstrates high diagnostic frequencies but also carries a substantial mortality burden [3]. Its incidence varies across regions, affecting approximately 13.3 million people worldwide every year, with a mortality rate that remains notably high [4]. Several factors contribute to the development of AKI, including severe infection, hypovolemia, nephrotoxic drug exposure, and postoperative complications [5, 6]. A deeper understanding of its pathophysiology highlights a complex interplay between inflammation, oxidative stress, and altered renal blood flow, cumulatively resulting in renal tissue damage [7, 8]. Early detection and prompt intervention are crucial for preventing further renal deterioration and improving patient prognosis [9]. Although current AKI treatment focuses primarily on supportive measures such as fluid replacement, hemodynamic stabilization, and addressing underlying causes, the prognosis of the disease remains concerning even with advanced critical care [10, 11]. This stark reality emphasizes the pressing need for novel diagnostic biomarkers, innovative therapeutic approaches, and predictive tools to enhance early AKI detection, timely management, and comprehensive care.

AKI presents a formidable clinical conundrum, the intricate molecular facets of which remain elusive. Delving into the genetic intricacies of AKI, the TCF4 prominently stands out [12, 13]. This gene is essential for the differentiation and proliferation of cells, and it is also essential for the repair and regeneration of renal tissue [14, 15]. Dysregulation of TCF4 not only casts a shadow over the cellular dynamics but can also critically impede innate renal recovery post-injury [16]. Conversely, COX7A2L, although not directly associated with AKI, warrants attention due to its paramount significance in cellular energy metabolism and in ensuring mitochondrial robustness [17, 18]. Furthermore, the influence of COX7A2L on the electron transport chain makes it central to cellular respiration and energy production, factors that are crucial when considering the metabolic demands of injured renal cells [19, 20]. Even without a direct link to renal pathology, the multifaceted functions of COX7A2L cannot be overlooked, positioning it as a potential candidate for further exploration in AKI research. Given the formidable challenges that AKI poses, a comprehensive understanding of both COX7A2L and TCF4 emerges as a pivotal step, potentially revolutionizing diagnostic precision and therapeutic interventions.

Recognizing the urgent need for an enhanced understanding of AKI and innovative therapeutic strategies, this study aims to explore the intricate interaction between COX7A2L and TCF4 and its possible involvement in the pathophysiology of AKI. By delving deeply into the GSE139061 and GSE30718 datasets, our study identified key genetic modules and their key associations with AKI, specifically highlighting the profound correlation between the turquoise module and AKI. Central to our research is the elucidation of the effects of COX7A2L on cellular processes such as apoptosis and proliferation, especially under H/R conditions, as well as the regulatory role of TCF4 in COX7A2L transcription. Through this comprehensive exploration, we aim to uncover novel molecular mechanisms driving AKI progression, provide promising diagnostic markers, and lay the foundation for targeted therapeutic intervention in the management of AKI.

## Materials and methods

### Dataset collection

A database named Gene Expression Omnibus (GEO) (https://www.ncbi.nlm.nih.gov/geo/) provided the datasets GSE139061 and GSE30718. 39 samples of AKI and 9 samples of normal were obtained from the GSE139061 dataset, and this dataset was profiled using the GPL20301 Illumina HiSeq 4000 (Homo sapiens) platform. Conversely, the GSE30718 dataset utilized the GPL570 [HG-U133_Plus_2] Affymetrix Human Genome U133 Plus 2.0 Array, comprising 28 samples from patients with AKI and 11 samples from individuals with normal renal function. The selection of these samples was made specifically due to their association with AKI and was used for subsequent analysis in this study.

### Analysis of differential gene expression in the GSE139061 dataset

With the purpose of differential expression analysis, we specifically utilized the GEO2R tool to analyze the GSE139061 dataset. The identification of differentially expressed genes (DEGs) in AKI and control samples was made easier by this method. To pinpoint the upregulated and downregulated genes, we employed specific criteria: Downregulated DEGs were identified by a *p*-value < 0.05 and a fold change (FC) < 0.77, whereas upregulated DEGs were identified by a *p*-value < 0.05 and an FC > 1.3.

### Weighted gene co-expression network analysis (WGCNA)

To uncover co-expression patterns among DEGs from the GSE139061 dataset, we employed the WGCNA using the dedicated R package. First, a sample clustering tree is constructed to detect and remove any outlier samples. Next, Pearson correlation analysis was applied to calculate the adjacency matrix for all genes. For a more thorough analysis of gene-gene connections, use a suitable soft-threshold value. Gene connections generated in the network follow a scale-free distribution. Subsequently, the outcomes are then converted into a topological overlap matrix (TOM) by moving them to an adjacency matrix. To determine interesting modules connected to AKI, correlations between module eigengenes and AKI were calculated using Pearson correlation analysis. The interest module was chosen as the one with the strongest association with AKI, indicating a potential functional relevance to the disease.

### Functional enrichment and PPI network analysis of key module

The Database for Annotation, Visualization, and Integrated Discovery (DAVID, https://david.ncifcrf.gov/tools.jsp) is a frequently employed bioinformatics resource for the functional annotation of gene lists generated from high-throughput experiments [21]. The database provides a variety of functions, including gene function classification, functional annotation chart, and functional annotation clustering. In this study, DAVID was utilized to carry out pathway enrichment analysis of Gene Ontology (GO) and Kyoto Encyclopedia of Genes and Genomes (KEGG) for important modules found by WGCNA in the GSE139061 dataset. Furthermore, the Search Tool for the Retrieval of Interacting Genes (STRING) database (https://string-db.org/) was used to conduct PPI network analysis for significant module genes. The Cytoscape program was used to visualize and improve this PPI network. Then, important genes were found and their interactions were shown using the Molecular Complex Detection (MCODE) method of the CytoHubba plugin.

### Validation of candidate gene expression levels and receiver operating characteristic (ROC) analysis

To validate the significance of our discoveries, we assessed the gene expression levels using the MCODE algorithm in the GSE139061 and GSE30718 datasets, yielding statistically significant results at *p*<0.05. Among these genes, four demonstrated notable differential expression across both datasets. Subsequently, we conducted a ROC analysis for each of these 15 genes, separately within the two datasets. We evaluated the possible clinical diagnostic usefulness of these genes in the setting of AKI by calculating the area under the curve (AUC) value.

### Cell culturing and hypoxia/reoxygenation (H/R) model establishment in NRK-52E cells

NRK-52E cells, a rat renal proximal tubular cell line, were sourced from the Cell Bank of the Chinese Academy of Sciences (Shanghai, China). These cells were maintained in DMEM/F12 medium supplemented with 10% fetal bovine serum (FBS) and 1% penicillin-streptomycin (PS) at 37°C under a 5% CO_2_ humidified atmosphere. To simulate the hypoxia/reoxygenation (H/R) model, NRK52E cells was induced by 24 hours of hypoxia (94% N₂ + 1% O₂ + 5% CO₂, glucose and serum-free DMEM/F12 medium) followed by reoxygenation for 1 hour, 3 hours, 6 hours, 9 hours, or 12 hours (95% air and 5% CO₂, DMEM/F12 medium with 10% FBS) [22].

### Cell transfection

A density of 2 × 10^5^ cells per well was used to seed NRK-52E cells in 24-well plates for transient transfection. NRK-52E cells were transfected individually with the plasmids expressing COX7A2L and TCF4, utilizing the proper transfection techniques. The transfected cells were allowed to express COX7A2L protein and TCF4 protein within a specific period to achieve overexpression. Then, NRK-52E cells were transfected with a particular small interfering RNA (siRNA) (which the use of scrambled siRNA as a control) that targets COX7A2L to decrease COX7A2L expression, and cells were incubated for a specific time to allow efficient knockdown of COX7A2L. Using Lipofectamine 3000 (Invitrogen, USA), transfections were carried out in accordance with the manufacturer’s instructions.

### Quantitative real-time polymerase chain reaction (qRT-PCR) assay

The TRIzol reagent (Thermo Fisher Scientific, USA) was used to extract the total RNA of NRK-52E cells following the manufacturer’s instructions. We used a Takara, Japan-sourced PrimeScript RT kit for cDNA synthesis. The StepOnePlus Real-Time PCR System was used to run qRT-PCR with the SYBR Green PCR Master Mix from Applied Biosystems, USA. The 2^-ΔΔCT^ technique was used to examine the data, and β-actin abundance was used as a standard. The amplification procedure made use of the primer sequences listed below: COX7A2L primers: forward 5’-AGCCAAGCAATAAAAGAGTTCG-3’, reverse 5’-GGTCACCACGAAGGGTAAATG-3’. Similarly, primers for the reference gene GAPDH were as follows: β-actin forward 5’-CAAGCTCATTTCCTGGTATGAC-3’, β-actin reverse 5’-CAGTGAGGGTCTCTCCTTCCT-3’.

### Western blotting (WB) assay

Protein lysates from NRK-52E cells were obtained using RIPA lysis solution (Thermo Fisher Scientific, USA), supplemented with phosphatase and protease inhibitors. A protein assay kit from Thermo Fisher Scientific (USA) was used to measure the protein content. The proteins were then transferred onto PVDF membranes (Millipore, USA) after being separated using SDS-PAGE. Primary antibodies against β-catenin, c-myc, cyclin D1, active caspase 3, TCF4, BAX, Bcl-2, COX7A2L, and Bcl-2 (all from Abcam, USA) were used at a 1:1000 dilution. β-actin (1:5000, Abcam, USA) served as the loading control. Thermo Fisher Scientific’s enhanced chemiluminescence (ECL) kit was used to observe the protein bands, and a ChemiDoc system (Bio-Rad, USA) was used to record the images.

### Flow cytometry

NRK-52E cells were detached using trypsin-EDTA (Gibco, USA) and then washed with phosphate-buffered saline (PBS) in preparation for flow cytometric analysis. Following the manufacturer’s directions, stain cells with Annexin V and propidium iodide (PI) to distinguish between live, apoptotic, and necrotic cells. Cell apoptosis was assessed using flow cytometry with equipment from BD Biosciences, USA, and the results were processed with FlowJo software.

### Cell counting kit-8 (CCK-8) assay

The viability of the cells was assessed using the CCK-8 assay (Dojindo, Japan). A density of 5 × 10^3^ cells per well was used to plant NRK-52E cells in 96-well plates. Each well was supplemented with CCK-8 reagent and allowed to incubate for an additional 1, 2, 3, 4, and 5 days, respectively. Cell vitality was then assessed by recording absorbance at 450 nm with a microplate reader (Thermo Fisher Scientific-USA).

### Luciferase reporter assay

The pGL3-Basic vector, which carries the mutant (MUT) or wild-type (WT) COX7A2L promoter sequence, was co-transfected with the pRL-TK plasmid (Renilla luciferase) in HEK 293T cells. Furthermore, cells were transfected with a vector expressing TCF4 (over-TCF4) or an empty vector control (negative control). A dual-luciferase reporter assay method was used to measure the activities of both firefly and Renilla luciferases 48 hours after transfection Transfection efficiency was adjusted by normalizing firefly luciferase activity to Renilla luciferase.

### Chromatin immunoprecipitation (ChIP) assay

ChIP experiments were performed to look into TCF4’s possible binding to the COX7A2L promoter in HEK 293T cells. In the procedure, HEK 293T cells underwent cross-linking with 1% formaldehyde. Subsequently, the chromatin was extracted and sheared into fragments ranging from 200–500 bp through sonication. For immunoprecipitation, the sheared chromatin was treated with either an anti-TCF4 antibody or an IgG control (we used irrelevant IgG as a negative control to verify antibody specificity), protein A/G Plus Agarose beads were then added and incubated. After reversing the cross-links, the DNA was purified and employed in qRT-PCR assays using primers specific for the COX7A2L promoter region. The fold enrichment of the COX7A2L promoter in samples treated with the TCF4 antibody was determined by comparing its levels to those in the IgG control samples.

### Statistical analysis

The R programming language was employed for data analysis. The statistical analysis was conducted with GraphPad Prism 8.0. Student’s t-test assessed intergroup differences. The mean ± standard deviation (SD) is used to show all data and one-way ANOVA is used for analysis before Tukey’s post hoc test is performed. Each experiment was replicated at least three times in our study. It was considered statistically different when *p* was at two-tailed < 0.05 (*p < 0.05, ** *p* < 0.01, *** *p* < 0.001).

## Results

### The Turquoise module is highly correlated with AKI samples in GSE139061

**Fig 1:**
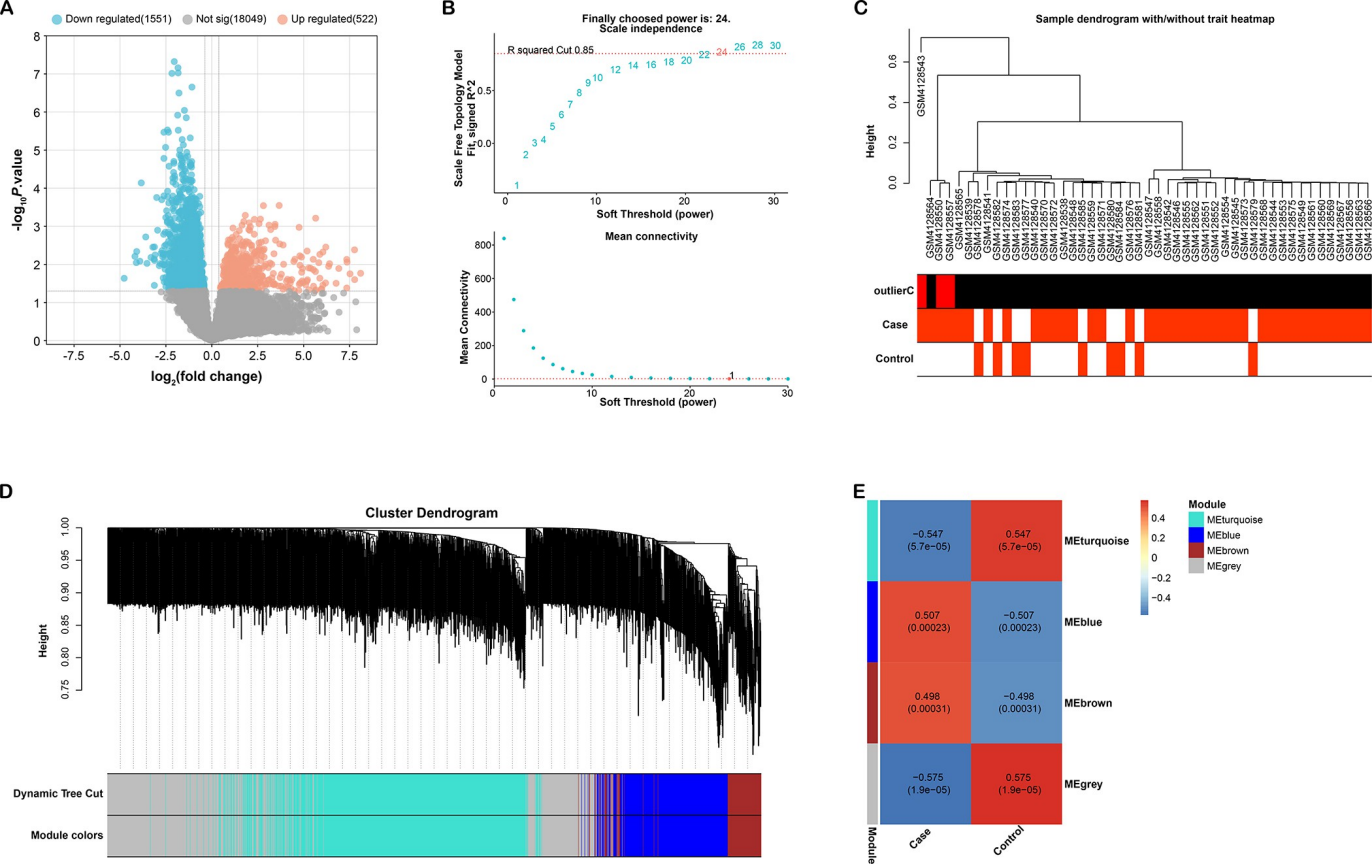
Screening of DEGs and weighted gene co-expression network analysis. (A) Volcano plot depicting the results of differential gene expression analysis. Orange represents up-regulated DEGs, and blue represents down-regulated DEGs. (B) The upper figure shows the determination of the optimal soft threshold in the gene co-expression network, and the lower figure shows the mean connectivity for different soft thresholds. Scale-free distribution is a concept in network science that describes the fact that the node degree (number of connections) of certain networks follows a power law distribution, R^2^ value is the coefficient of determination to assess the superiority of the fitted model (scale-free R^2^ = 0.85, power = 24). (C-D) Cluster dendrogram of gene modules with different colors. (E) Heatmap of the correlation between gene modules and GSE139061 samples, the numbers in the modules represent the correlation coefficients and *p*-values.

In the GSE139061 dataset, the DEGs were visualized using the R package (Fig 1A), unveiling 522 upregulated and 1551 downregulated DEGs. To elucidate the interconnectivity among these DEGs, a gene network exhibiting a scale-free distribution (which is a concept in network science that describes the fact that the node degree (number of connections) of certain networks follows a power law distribution) was constructed, R^2^ value is the coefficient of determination, which is used to assess the superiority of the fitted model (scale-free R^2^ = 0.85, power = 24) (Fig 1B). To bolster the credibility of the findings, a sample clustering analysis was conducted, targeting the identification and elimination of potential outlier samples (Fig 1C). Following this, a hierarchical clustering based on gene dissimilarity (1-TOM) delineated four discrete modules (Fig 1D). Each of these modules was represented by a module eigengene. The relationship between these modules and AKI was assessed using module signature relationship analysis. Significantly, a profound correlation was identified between the turquoise module and AKI (r = -0.547, *p* = 5.7e-05), pointing to its probable role in the pathogenesis of AKI (Fig 1E).

### Functional enrichment analysis reveals key processes and pathways associated with turquoise module genes

**Fig 2:**
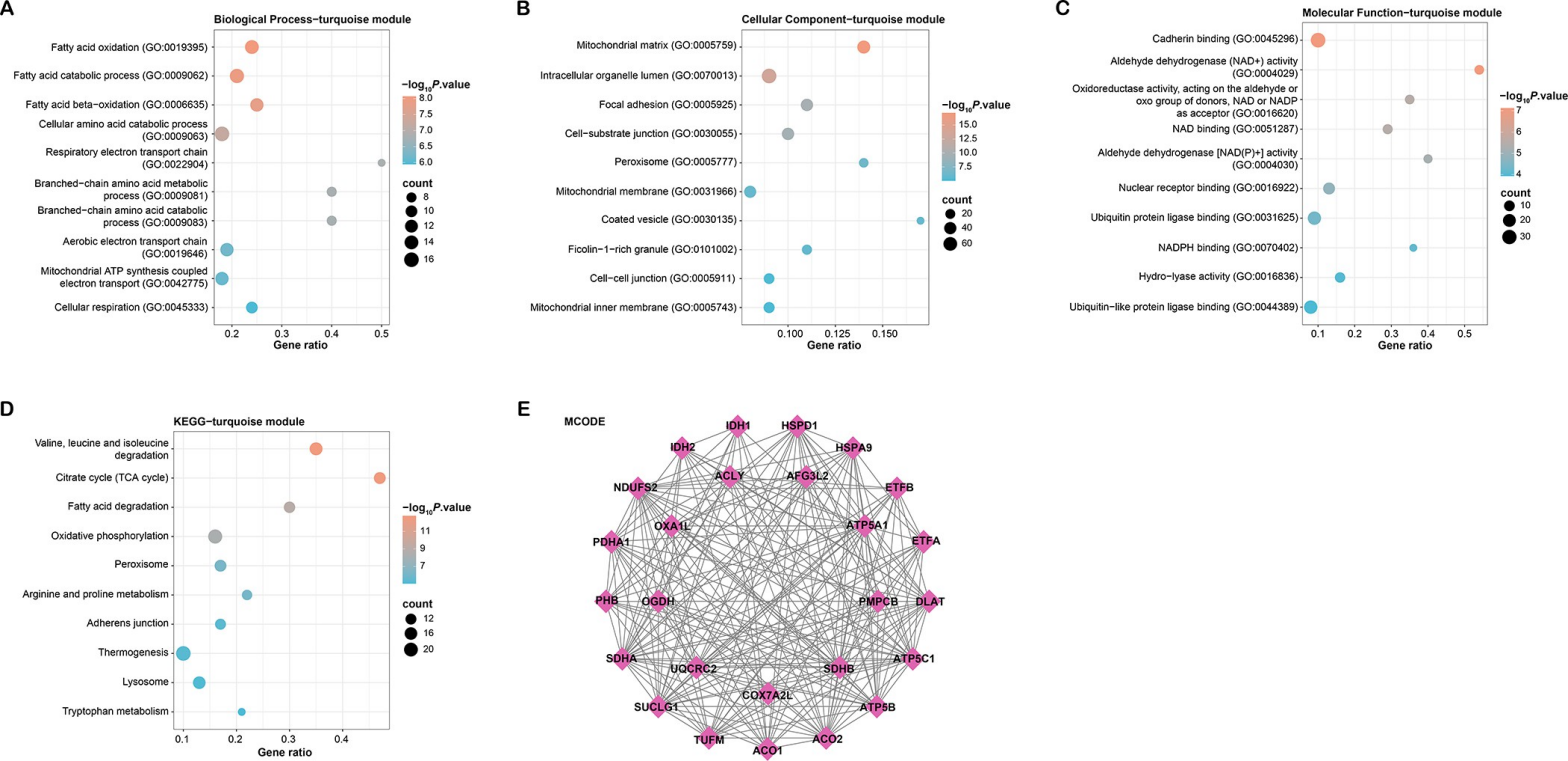
Functional annotation and PPI analysis of turquoise module genes. (A-D) GO and KEGG enrichment analysis of turquoise module genes. The X-axis represents the Gene Ratio; the Y-axis represents the GO Term or enriched pathway; the size of the dots represents the odds ratio; the color of the dots represents the level of the *p*-value. (E) PPI network of turquoise module genes using the MCODE algorithm, with nodes represent proteins or protein domains, while edges represent interactions between these proteins.

The genes within the turquoise module (S1 Table) were found to be significantly enriched in several biological processes, including fatty acid oxidation, peroxisomes, the mitochondrial matrix, cadherin binding, and aldehyde dehydrogenase (NAD+) activity, according to the results of the GO enrichment analysis (Fig 2A–2C). These enrichments indicate that the genes of the turquoise module are important regulators of intracellular compartmental structure, molecular interactions, and fatty acid metabolism. Genes in the turquoise module are enriched in multiple KEGG pathways, including the peroxisome, adherens junction, lysosome, and tryptophan metabolism pathways (Fig 2D). These findings highlighted the involvement of the turquoise module genes in energy metabolism, specifically in processes related to mitochondrial function and lipid metabolism. PPIs in the turquoise module gene were further explored using the MCODE algorithm (S2 Table). MCODE extracted a core gene module in PPI, containing 26 nodes and 221 edges (Fig 2E).

### Differential expression of key genes in AKI reveals potential diagnostic markers

**Fig 3:**
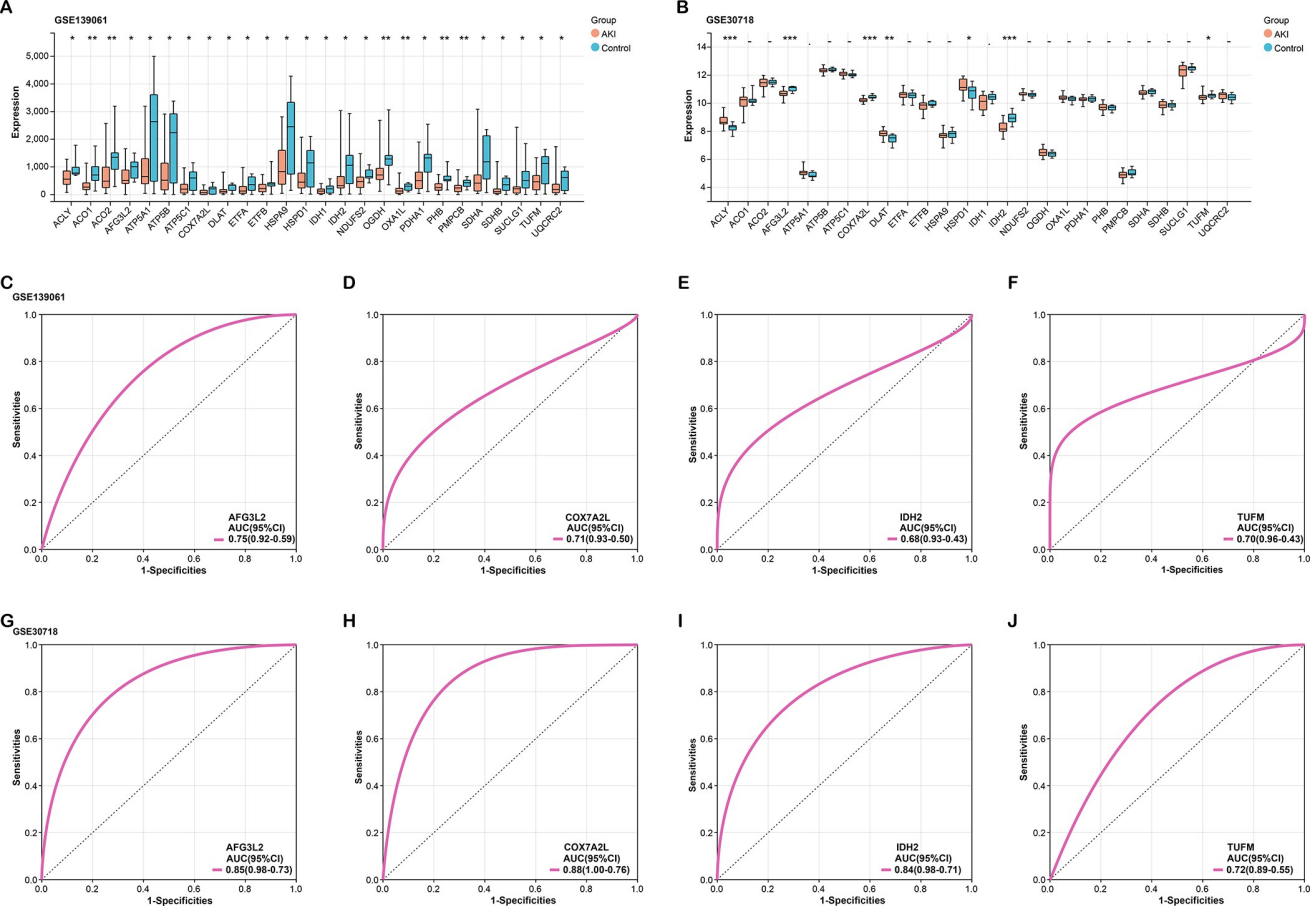
Expression analysis and diagnostic potential of 26 candidate genes in the AKI Turquoise module. (A) Expression analysis of 26 candidate genes in AKI samples compared to control samples using the GSE139061 dataset. (B) Validation of the expression patterns of 26 candidate genes in AKI samples using the GSE30718 dataset. (C-F) ROC curves describing the diagnostic potential of candidate genes based on the GSE139061 dataset (C: AFG3L2, D: COX7A2L, E: IDH2, F: TUFM). (G-J) ROC curves illustrating the diagnostic potential of candidate genes based on the GSE30718 dataset (G: AFG3L2, H: COX7A2L, I: IDH2, J: TUFM). *p<0.05. ** *p*<0.01, *** *p*<0.01.

To assess the expression profile of the 26 nodal genes within the turquoise module in AKI, we analyzed the GSE139061 dataset. As compared to control samples, the outcomes demonstrated that these nodal genes were considerably downregulated in AKI samples (Fig 3A). To confirm the expression of the 26 genes even further, we analyzed the GSE30718 dataset and observed that there were 7 genes with significant expression differences in GSE30718. Four of them were low expressed in both GSE139061 and GSE30718 (including AFG3L2, COX7A2L, IDH2, TUFM), so they were selected for ROC curve analysis (Fig 3B). The ROC curves for the GSE139061 dataset (Fig 3C–3F) and the GSE30718 dataset (Fig 3G–3J) demonstrated the discriminative power of the selected genes. Notably, COX7A2L and AFG3L2 exhibited significant diagnostic value, as evidenced by high AUC values in both datasets. Since COX7A2L has not been reported in acute kidney injury, we chose it as a hub gene for further analysis.

### COX7A2L modulation of apoptosis and proliferation in cells subjected to H/R treatment

**Fig 4:**
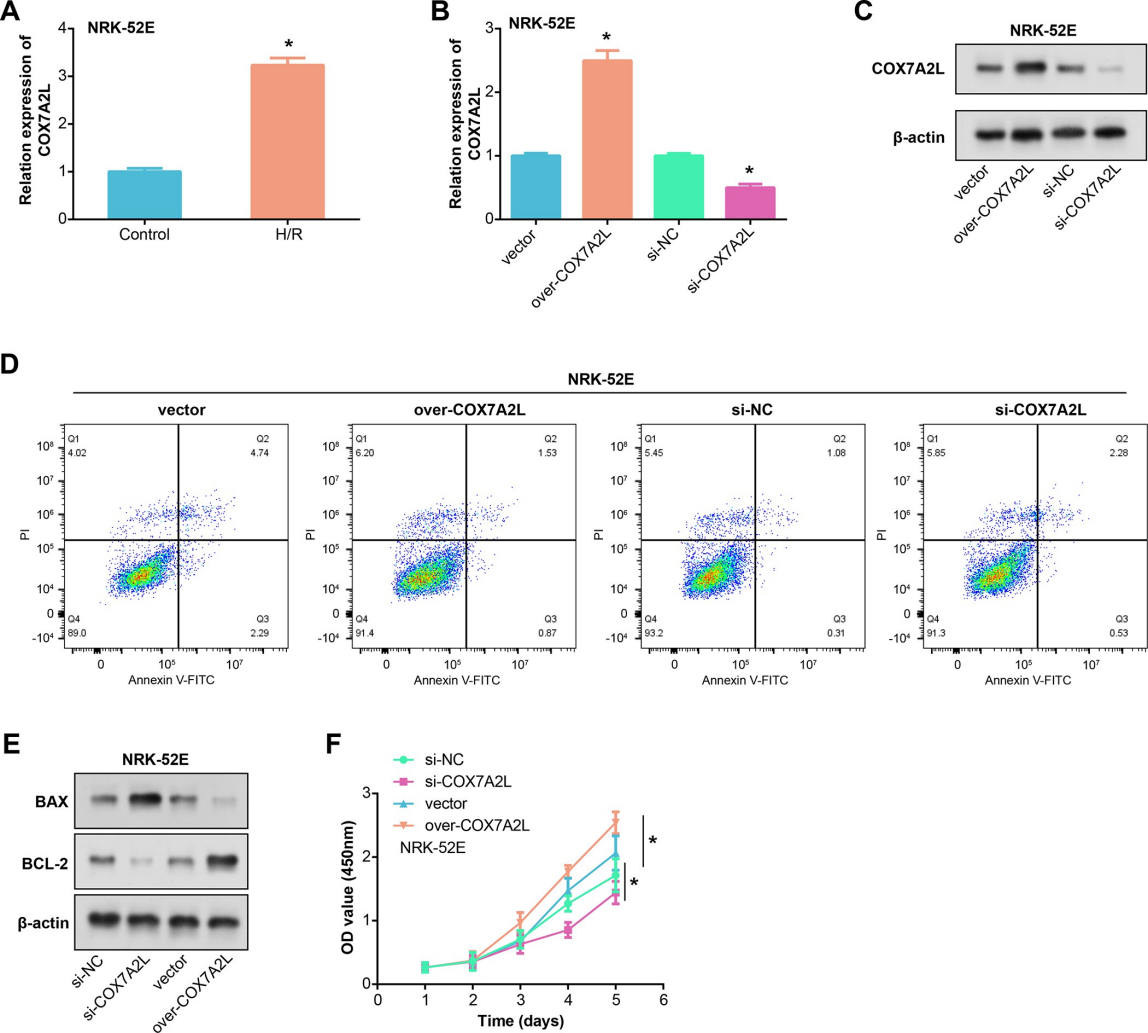
COX7A2L inhibits H/R-treated NRK-52E cell apoptosis and promotes cell proliferation. (A) qRT-PCR detects the expression of COX7A2L in NRK-52E cells after H/R treatment. (B-C) qRT-PCR and WB detected the overexpression efficiency and knockdown efficiency of COX7A2L. (D) Flow cytometry to detect the apoptotic effects of COX7A2L knockdown and overexpression on H/R-treated NRK-52E cells. (E) WB analysis of the effect of COX7A2L on the expression of pro-apoptotic protein Bax and anti-apoptotic protein Bcl-2 in H/R-treated cells. (F) CCK-8 analysis of the effects of COX7A2L knockdown or overexpression on cell proliferation. *p<0.05.

The results of the qRT-PCR study indicated that, in comparison to the control group, the H/R group’s expression of COX7A2L was reduced (Fig 4A). To confirm the effectiveness of COX7A2L overexpression and knockdown in NRK-52E cells, both WB and qRT-PCR tests were used, as shown in Fig 4B and 4C. Specifically, COX7A2L overexpression led to a notable augmentation in its expression, while its knockdown caused a pronounced reduction. Flow cytometry findings indicated that overexpression of COX7A2L decreased apoptosis in H/R-treated cells, whereas knockdown of COX7A2L accelerated death in H/R-treated cells (Fig 4D). A pro-apoptotic protein called Bax is important in controlling the stages of apoptosis execution and initiation [23]. In contrast, Bcl-2 is an anti-apoptotic protein that can stop apoptosis from happening [24]. WB analysis, as shown in Fig 4E, indicated that COX7A2L was able to inhibit apoptosis. Furthermore, CCK-8 assays demonstrated that altering COX7A2L levels had a significant impact on cell proliferation, with knockdown suppressing it, and overexpression promoting it (Fig 4F).

### TCF4 enhances COX7A2L transcription

**Fig 5:**
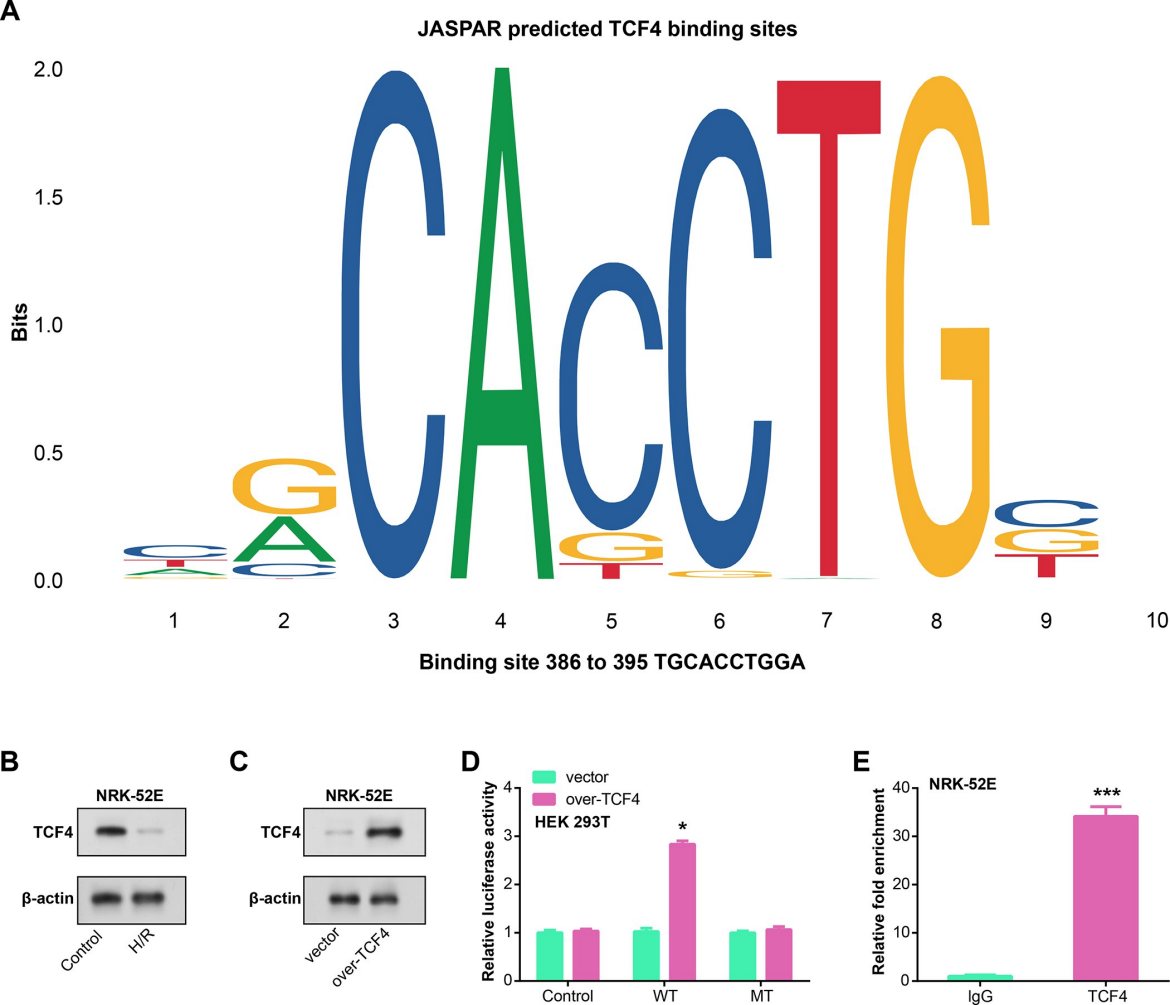
TCF4-mediated regulation of COX7A2L expression. (A) JASPAR database analysis reveals putative TCF4 binding sites in the promoter region of differentially expressed genes. (B) WB analysis of TCF4 protein expression in H/R-treated NRK-52E cells. (C) WB analysis of TCF4 overexpression efficiency in H/R-treated NRK-52E cells. (D) Luciferase reporter assay results in HEK 293T cells. Constructs harboring either the wild-type (WT) or mutant (MT) COX7A2L promoter sequences were utilized. (E) ChIP assay of TCF4 binding to COX7A2L promoter, IgG as negative control. *p<0.05, ***p<0.001.

Identify a set of differentially expressed genes through querying the JASPAR database, and subsequently utilize this subset to predict the presence of TCF4 binding sites within the promoter regions associated with these genes (Fig 5A). When compared to the control group, WB analysis showed that the H/R group’s TCF4 protein expression was considerably down-regulated, indicating that TCF4 may be involved in the regulation of COX7A2L (Fig 5B). To further investigate the functional role of TCF4, the overexpression efficiency in H/R-treated rat proximal tubular epithelial cells by WB analysis, confirmed successful TCF4 overexpression (Fig 5C). To investigate the direct transcriptional control of COX7A2L by TCF4, luciferase reporter experiments were conducted utilizing reporter constructs incorporating wild-type (WT) or mutant (MUT) COX7A2L promoter sequences. The findings showed that TCF4 overexpression greatly affected the reporter gene’s activity that carried the COX7A2L promoter WT sequence (Fig 5D), suggesting that TCF4 and the COX7A2L promoter may interact. To further validate the interaction between TCF4 and the COX7A2L promoter, ChIP assays were conducted. The ChIP results confirmed the binding of TCF4 to the COX7A2L promoter, supporting its role as a potential transcriptional regulator of COX7A2L (Fig 5E).

### COX7A2L-dependent Wnt/β-catenin signaling promotes H/R-induced NRK-52E cell proliferation

**Fig 6:**
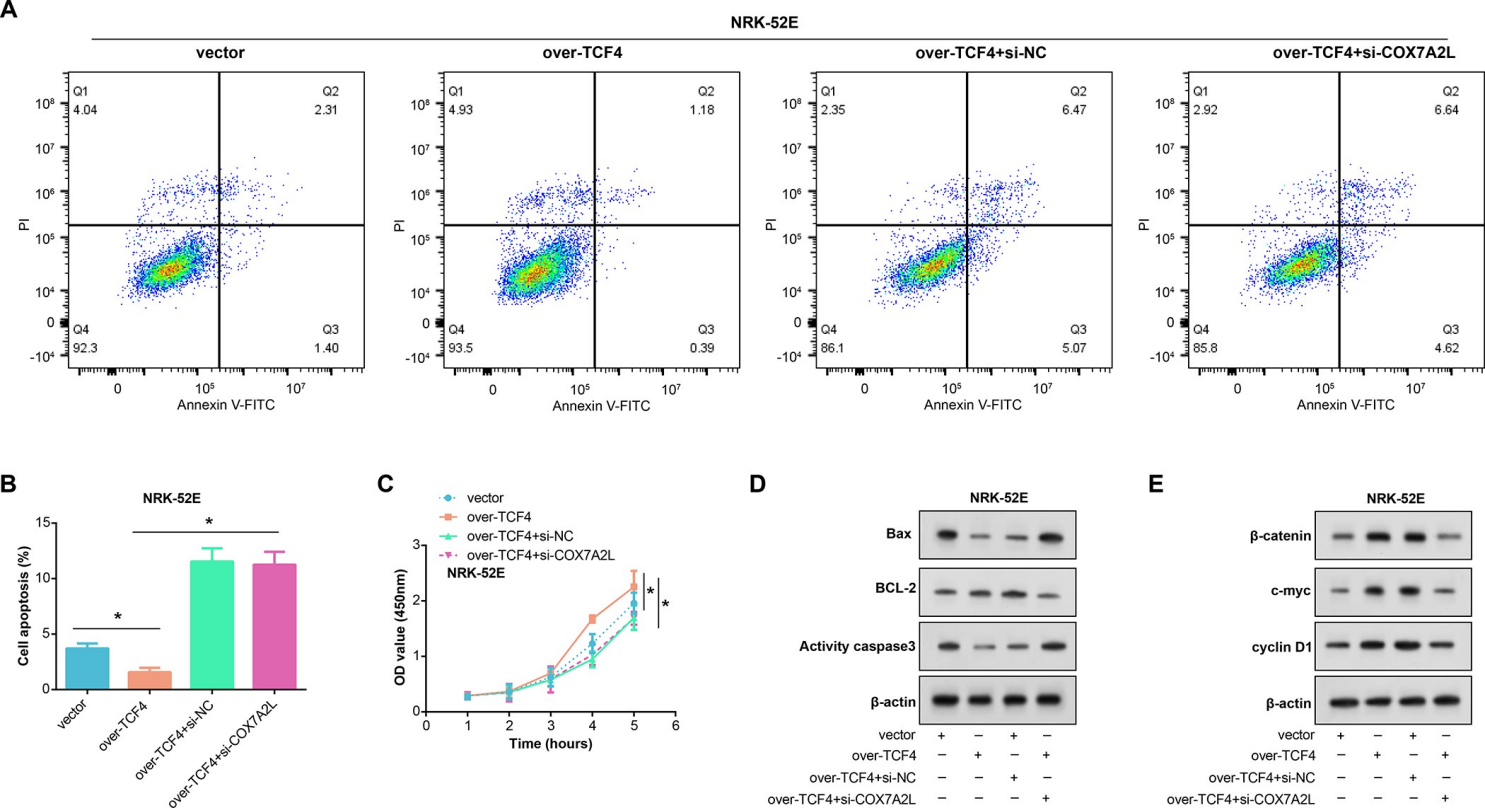
Effects of TCF4 overexpression and COX7A2L Silencing on apoptosis, proliferation, and Wnt/β-catenin signaling pathway in NRK-52E cells. (A-B) Flow cytometry analysis of apoptotic changes in NRK-52E cells after the following groups of treatments: vector, over-TCF4, over-TCF4+si-NC, over-TCF4+si-COX7A2L. (C) CCK-8 assay assesses NRK-52E cell proliferation in the following groups: vector, over-TCF4, over-TCF4+si-NC, over-TCF4+si-COX7A2L. (D-E) WB analysis of the regulation of apoptosis-related proteins (Bcl-2, Bax, caspase3) and Wnt/β-catenin signaling pathway proteins in H/R-treated NRK-52E cells by the following groups: vector, over-TCF4, over -TCF4+si-NC, over-TCF4+si-COX7A2L. *p<0.05.

When NRK-52E cells were exposed to H/R, flow cytometry analysis showed that TCF4 overexpression significantly reduced the rate of cell death. Conversely, silencing COX7A2L exacerbated this apoptotic response (Fig 6A and 6B). The findings of the CCK-8 experiment also showed that TCF4 overexpression encouraged cell growth, while the downregulation of COX7A2L counteracted this pro-proliferative effect, leading to significant suppression of cellular growth (Fig 6C). WB was used to evaluate the modulation of apoptosis-associated proteins (Bcl-2, Bax, and activity-caspase3) and elements of the Wnt/β-catenin signaling pathway in H/R-treated NRK-52E cells to clarify the molecular basis of these data. In line with previous research, pro-apoptotic proteins Bax and caspase-3 were significantly reduced upon TCF4 overexpression, but anti-apoptotic protein Bcl-2 was significantly elevated. These effects were reversed upon COX7A2L knockdown (Fig 6D). Further, the overexpression of TCF4 significantly increased the levels of β-catenin, c-myc, and cyclin D1 expression. Fig 6E illustrated the stimulatory impact of TCF4 on the expression of β-catenin, c-myc, and cyclin D1 was reduced by the introduction of COX7A2L silencing.

## Discussion

AKI is a common and complex clinical syndrome with a particularly high incidence in hospitalised and critically ill patients. AKI is not only associated with higher morbidity and mortality, but may also lead to long term renal impairment, which can progress to chronic kidney disease (CKD) [25]. Renal function continues to deteriorate in some patients after the onset of AKI, which is associated with proximal tubular maladaptive repair due to a pro-inflammatory and pro-fibrotic milieu [26]. The clinical diagnosis and management of AKI mostly rely on biomarkers [27, 28]. Presently, AKI diagnosis predominantly hinges on clinical evaluations, such as fluctuations in serum creatinine levels and urine output [29]. These conventional markers, however, often fall short in sensitivity and specificity, posing diagnostic challenges and exacerbating patient outcome [30]. Nonetheless, comprehensive investigations remain warranted to ascertain the sustained efficacy and overarching benefits of targeted gene therapies in the AKI landscape.

We performed WGCNA analysis on the DEGs of the GSE139061 data set and obtained the key module, the turquoise module. Notably, genes of the turquoise module showed significant enrichment in various KEGG pathways and GO terms, emphasizing their possible role in the molecular mechanism and development of AKI. One salient KEGG pathway was the Tryptophan metabolism. Its relevance in AKI stems from its involvement in modulating inflammation and oxidative stress. As an illustration, the enzyme indoleamine 2,3-dioxygenase 1 (IDO1), pivotal in tryptophan metabolism, has been documented to influence immune responses, thereby exacerbating renal damage in AKI [31]. The Lysosome pathway also emerged as significant, with links to AKI via autophagy and lysosomal dysfunction [32]. Disturbances in autophagy and lysosomal function are correlated with AKI’s onset and progression. Diving into GO terms, Cellular respiration emerged as pivotal, emphasizing mitochondrial function’s role in AKI, where mitochondrial disturbances often precipitate renal cell damage [33]. Fatty acid oxidation, integral to renal energy metabolism, was another highlighted term [34]. Aberrations here have been linked with AKI, with potential renoprotective outcomes from targeting this metabolism. Treatment with human urogenic stem cells (USCs)-derived exosomes (USC-Exo) ameliorated renal injury and iron death in a mouse model of renal ischaemia/reperfusion injury (IRI)-induced AKI [35]. In essence, the GSE139061 dataset’s analysis unveiled genes crucial to AKI-related KEGG pathways and GO terms. These pathways, such as Tryptophan metabolism and Lysosome, among others, offer ripe areas for AKI research. Contemporary investigations continue to unravel their exact roles in AKI, guiding therapeutic advancements.

Through expression and ROC analysis, COX7A2L emerged as a pivotal gene exhibiting notable diagnostic merit in AKI. Known formally as cytochrome c oxidase subunit 7A2-like, COX7A2L is a nuclear-encoded protein integral to the mitochondrial respiratory chain complex IV [36]. Its vital role in orchestrating mitochondrial function and energy metabolism has been recognized [37]. The PERK-eIF2α-ATF4 axis increases expression of supercomplex assembly factor 1 (COX7A2L), promotes respiratory supercomplex (SC) assembly, and enhances mitochondrial respiratory function. Activation of PERK is sufficient to rescue the bioenergetic deficits induced by complex missense mutations in patients with mitochondrial diseases [38]. In muscle, increased COX7A2L expression was associated with reduced body fat and improved cardiorespiratory health. Furthermore, in mice, COX7A2L expression was specifically induced in muscle during exercise. These findings elucidate the genetic basis of mitochondrial supercomplex formation and function in humans and suggest that COX7A2L plays an important role in cardiorespiratory health [39]. These observations highlight the significance of COX7A2L in various physiological contexts and underscore its potential therapeutic implications. Recent insights into COX7A2L’s role in AKI development have been provided by various studies. For example, research conducted by Zhang et al. illustrated an elevated expression of COX7A2L in renal tubular epithelial cells of an AKI mouse model induced by ischemia-reperfusion injury [40]. Their findings further indicated that augmented COX7A2L expression intensifies mitochondrial dysfunction and renal damage, pointing to its potentially harmful impact on AKI onset. However, a contrasting perspective was offered by Li et al., who documented a protective effect of COX7A2L in AKI. In their study, COX7A2L expression diminished in renal tubular epithelial cells during AKI episodes. Through experiments, they found that enhancing COX7A2L expression could reduce oxidative stress, inflammation, and renal cell apoptosis, thereby attenuating the progression of AKI [41]. These contrasting findings highlight the multifaceted role of COX7A2L in AKI, whose effects appear to depend on different contexts and experimental parameters. Therefore, in-depth research is required to assess the viability of COX7A2L as a therapeutic target and to completely comprehend the mechanism by which it governs the development of AKI.

The functions of TCF4, also known as E2-2, and COX7A2L in the cellular response of renal proximal tubular epithelial cells to H/R damage were examined in this study. TCF4, a multifaceted transcription factor, is implicated in an array of processes including development, differentiation, neurodevelopment, and cancer [42]. Membrane-associated protein A2 (ANXA2) was found to induce β-collagen activation, which in turn triggers TCF4-induced transcription factor EB (TFEB). In addition, TFEB promotes lysosomal production and enhances autophagic flow, thereby attenuating AKI [43]. These mechanisms underscore the complex interplay between transcription factors and cellular stress responses. The study’s findings revealed an upregulation of COX7A2L in NRK-52E cells under H/R conditions, pointing to its potential involvement in adaptive cellular stress responses. This upregulation is pivotal for cellular homeostasis, as evidenced by increased apoptosis and suppressed cell proliferation upon COX7A2L knockdown. Such observations align with prior research emphasizing the role of COX7A2L in modulating apoptosis and cell proliferation across various cell types. Simultaneously, an elevation in TCF4 levels, a transcription factor linked with cell differentiation and proliferation, was observed under H/R conditions. The effects of TCF4 overexpression mirrored those of COX7A2L knockdown, hinting at a conceivable interplay between these entities during H/R injury.

After highlighting the functions of TCF4 and COX7A2L in H/R-induced cellular alterations, it is important to think about the larger molecular landscape, especially the Wnt/β-catenin signaling pathway. The Wnt/β-catenin pathway is an essential and highly conserved cellular signaling system that regulates several biological functions, such as cell division, death, and proliferation [44]. Wnt proteins attach to cell surface receptors when they are active, preventing β-catenin from being broken down and enabling it to build up in the cytoplasm. After that, β-catenin moves into the nucleus and controls target gene transcription there. TCF4 functions as a molecular switch in transcriptional control and is a nuclear effector in the Wnt/β-catenin signaling pathway [45]. The Wnt/β-catenin pathway has been notably addressed in recent AKI studies. According to research by Schunk SJ and others, the Wnt/β-catenin pathway has a variety of roles in kidney damage and subsequent healing [46]. Moreover, research from Guo X and the team indicated that IL-6 accelerates renal fibrosis following acute kidney injury by activating the Wnt/β-catenin pathway [47]. Given that TCF4 overexpression and COX7A2L inhibition resulted in the stimulation of the Wnt/β-catenin pathway and the increased production of the death marker caspase-3, it stands to reason that the pathway might have a critical role in H/R injury scenarios. This association further solidifies the potential therapeutic implications of modulating the Wnt/β-catenin pathway in AKI management. In light of these findings, the intricate roles of COX7A2L and TCF4, particularly concerning Wnt/β-catenin signaling, warrant more comprehensive exploration. Such in-depth understanding could pave the way for novel therapeutic interventions, targeting the nuanced molecular interplay, to mitigate and manage kidney injuries more effectively.

### Limitation

Although our study gained valuable insights into the potential role of COX7A2L in AKI, it is important to acknowledge that there are several limitations that may affect the interpretation and generalisation of our findings. First, our study is a preliminary exploration that we were unable to validate using other datasets or experimental techniques due to current resource and time constraints. This limits the robustness of our conclusions regarding the role of COX7A2L in AKI. In addition, the lack of experimental validation of human renal tissue samples, such as immunohistochemistry (IHC) or in situ hybridisation (ISH), limits our ability to confirm COX7A2L expression levels and localisation in a clinical setting. Finally, our study is only a preliminary exploration, and without further validation, the results may not be generalisable to different populations or settings. Thus limiting our understanding of the specific mechanisms by which COX7A2L affected AKI. The functional mechanisms by which COX7A2L plays a role in AKI remain unclear and require further investigation. Future research efforts should focus on addressing these limitations to improve the robustness and generalisability of our findings. This includes initiating further functional studies to elucidate the specific mechanisms by which COX7A2L is involved in AKI. By addressing these limitations and pursuing these avenues of research, we can gain a deeper understanding of the role of COX7A2L in AKI and further explore its potential impact on clinical practice.

## Conclusion

Our study revealed the functional relevance of COX7A2L in AKI and established its link with TCF4 and Wnt/β-catenin signaling. Notably, COX7A2L emerged as a potential diagnostic marker for AKI, with TCF4 enhancing its transcription. Furthermore, the promotion of H/R-induced NRK-52E cell proliferation was shown to be significantly aided by Wnt/β-catenin signaling that is reliant on COX7A2L. These findings revealed the potential of targeting COX7A2L and its related pathways for future therapeutic strategies in the treatment of AKI.

## Supporting information

S1 Table List of genes in the turquoise module. (XLSX)

S2 Table MCODE clustering of genes in turquoise module. (XLSX)

S1 File (ZIP)

S2 File (ZIP)
