# Supplementary figures and images for: TCF4 promotes apoptosis and Wnt/β-catenin signaling pathway in acute kidney injury via transcriptional regulation of COX7A2L
Source: PLoS One. 2024 Nov 5;19(11):e0307667. doi: 10.1371/journal.pone.0307667 (PMC11537394; doi:10.1371/journal.pone.0307667)

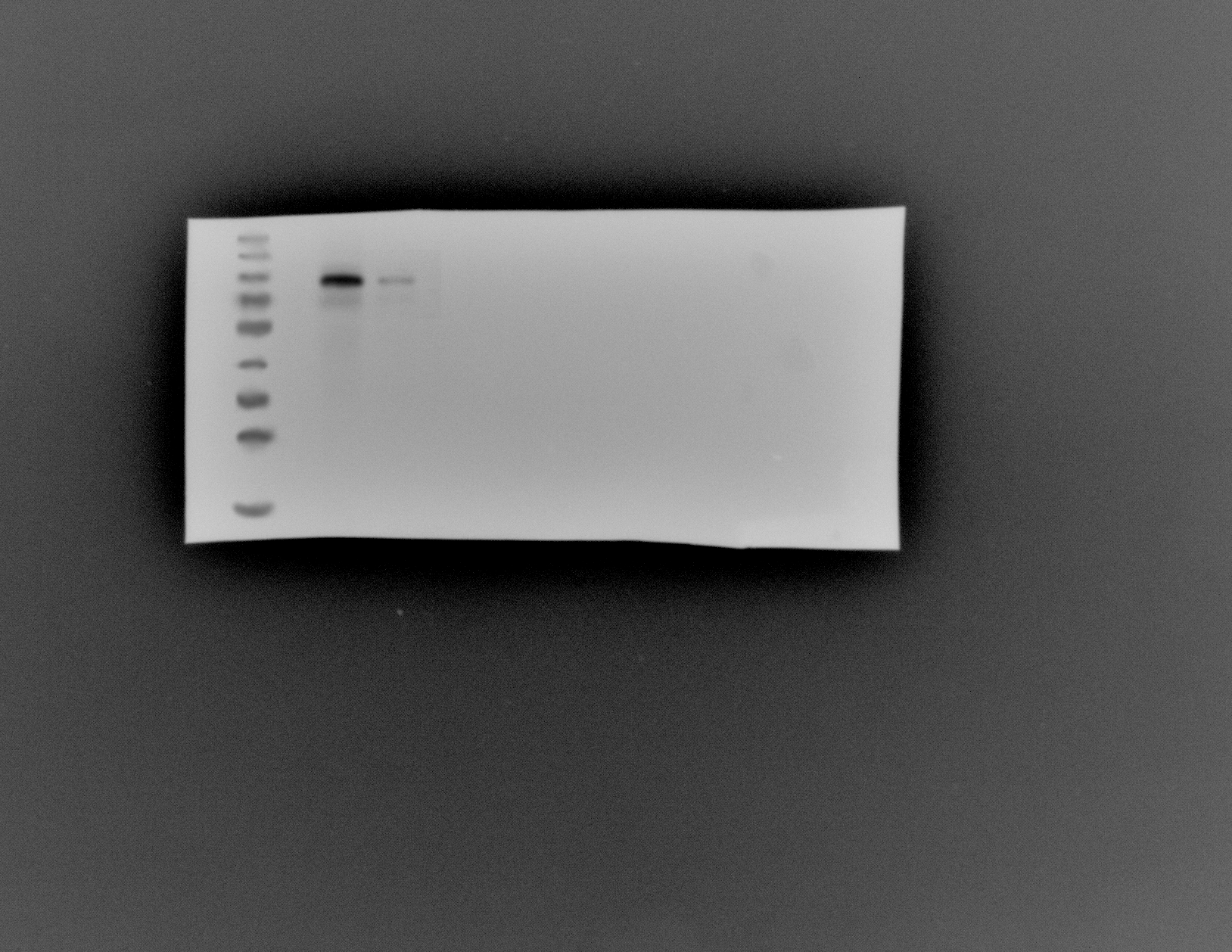

Supplement: S1 File — (ZIP) [file pone.0307667.s003.zip › figure 5- WB/Figure 5B_TCF4_NRK-52E.tif]

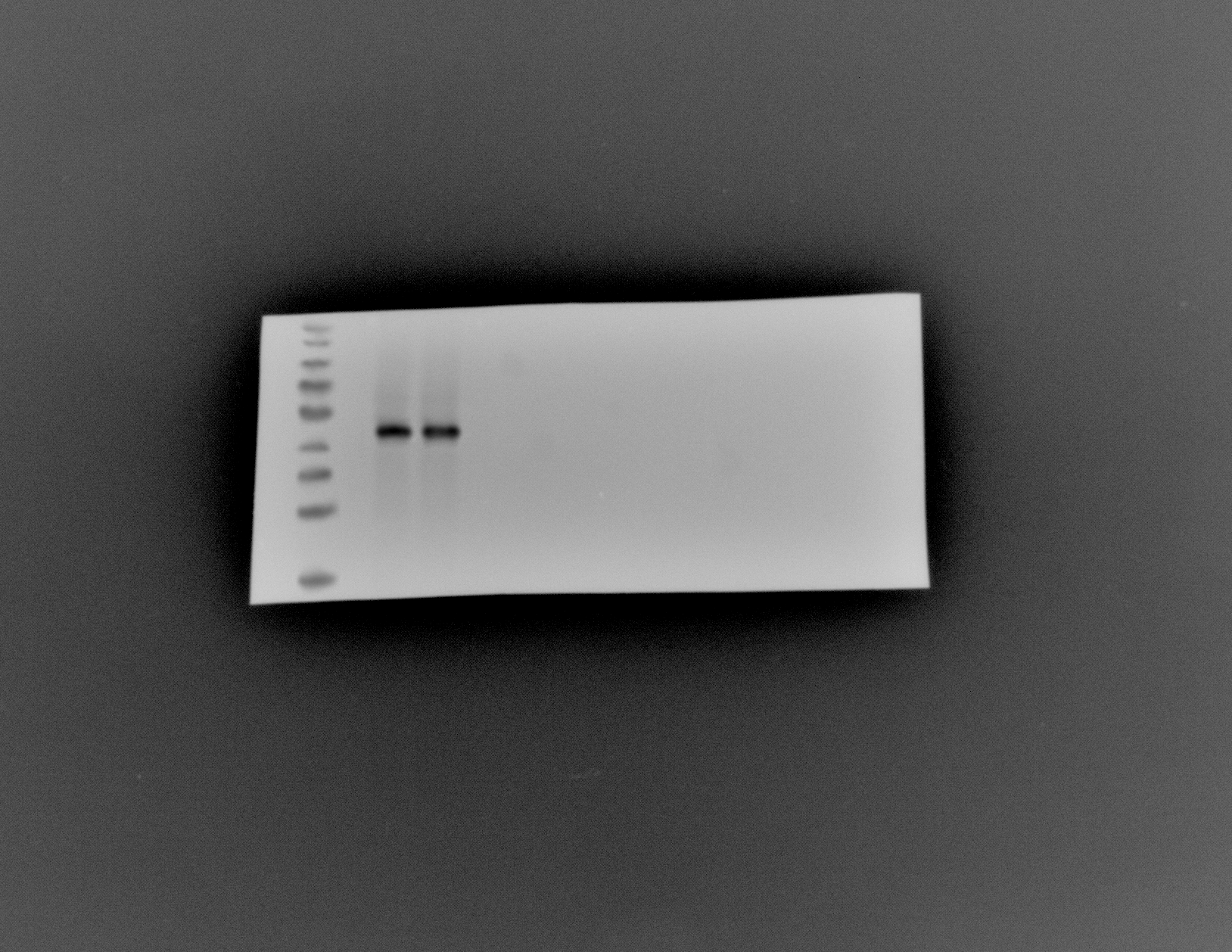

Supplement: S1 File — (ZIP) [file pone.0307667.s003.zip › figure 5- WB/Figure 5B_β-actin_NRK-52E.tif]

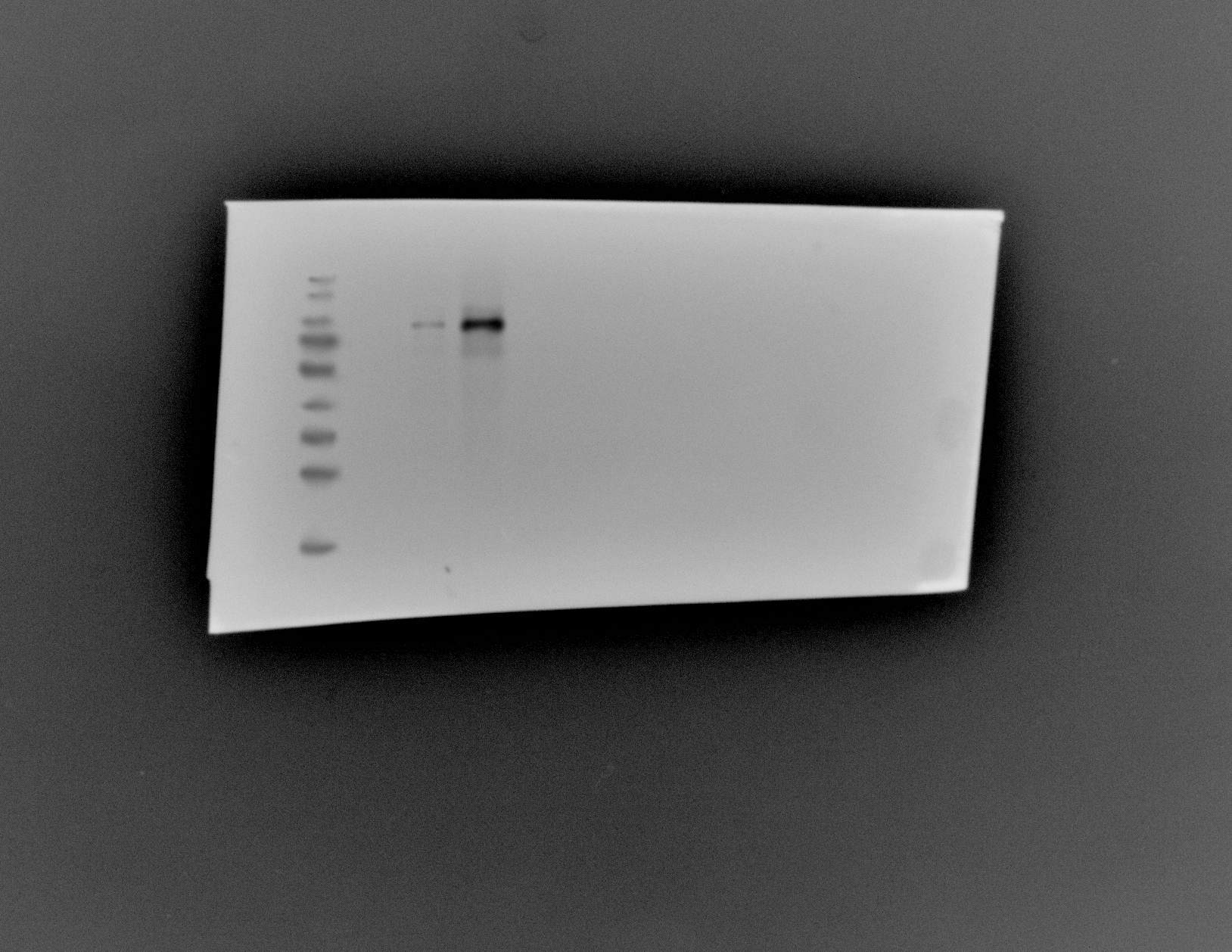

Supplement: S1 File — (ZIP) [file pone.0307667.s003.zip › figure 5- WB/Figure 5C_TCF4_NRK-52E.tif]

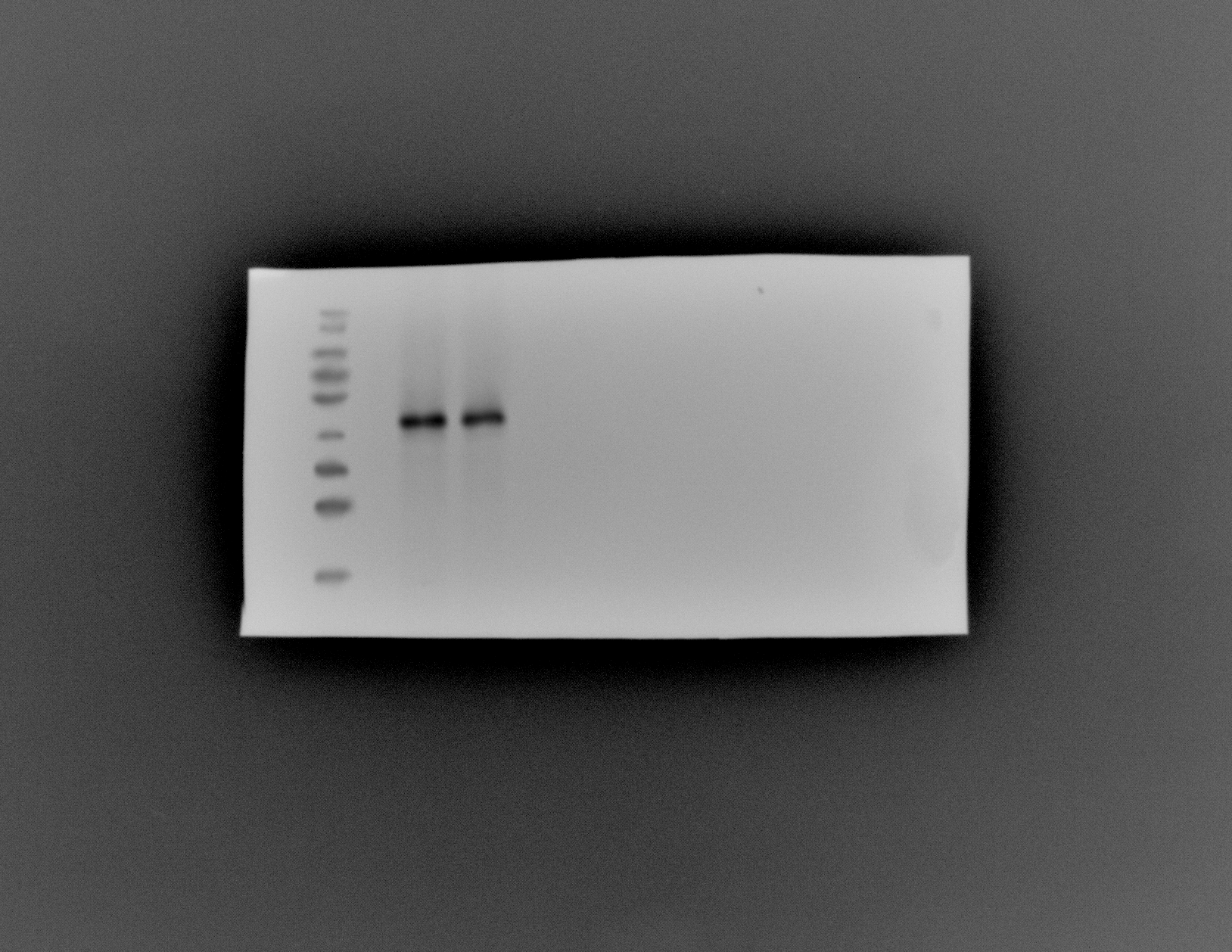

Supplement: S1 File — (ZIP) [file pone.0307667.s003.zip › figure 5- WB/Figure 5C_β-actin_NRK-52E.tif]

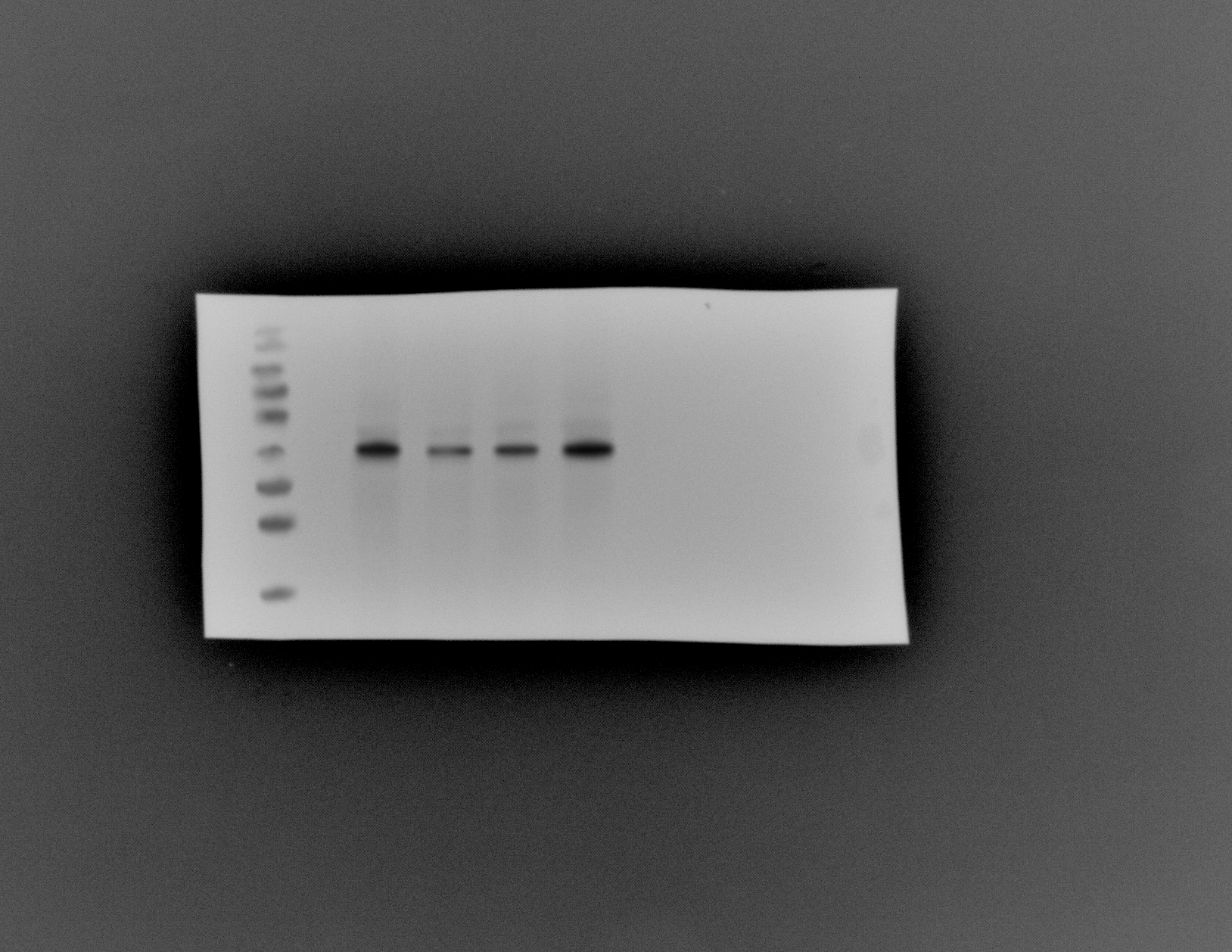

Supplement: S1 File — (ZIP) [file pone.0307667.s003.zip › figure 5- WB/Figure 5D_Activity caspase3_NRK-52E.tif]

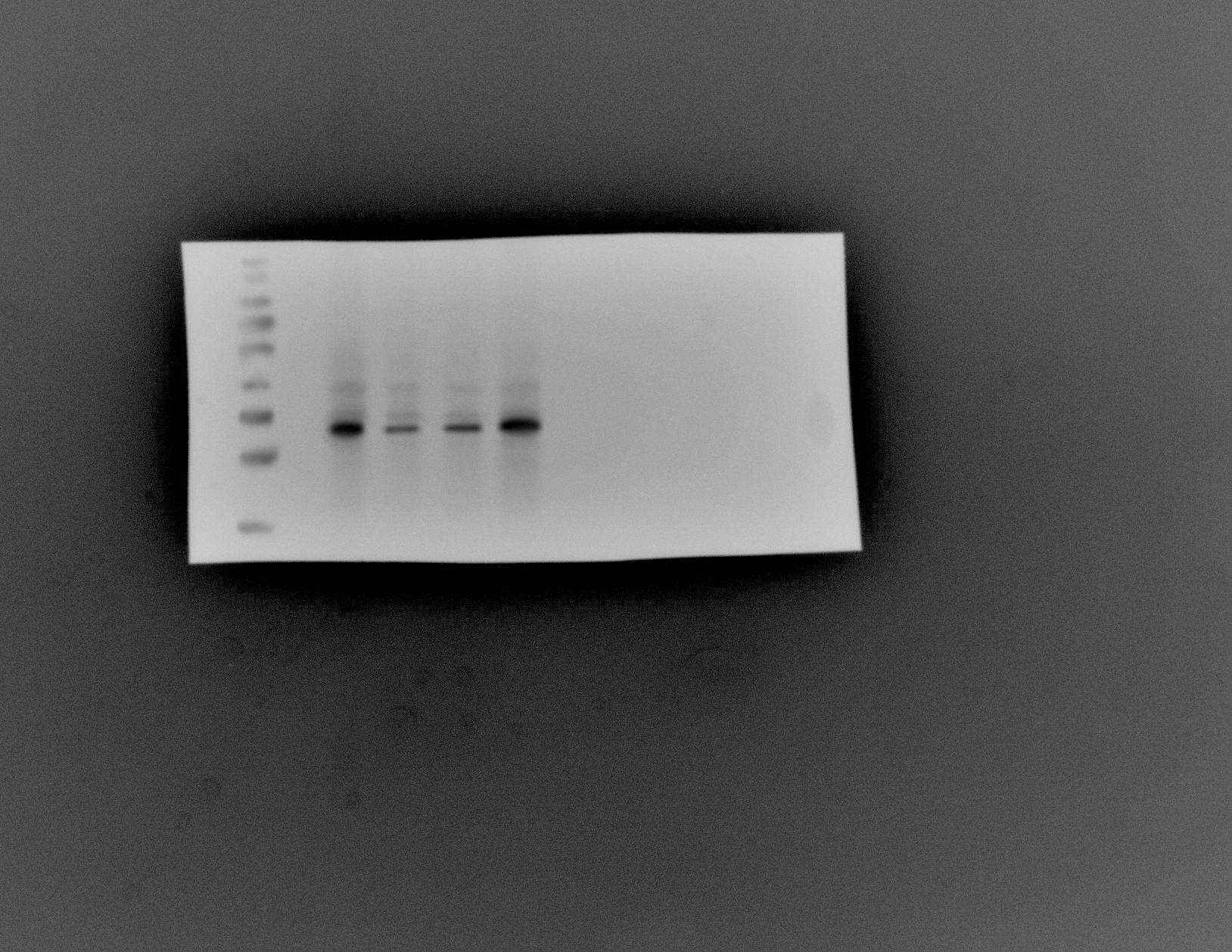

Supplement: S1 File — (ZIP) [file pone.0307667.s003.zip › figure 5- WB/Figure 5D_Bax_NRK-52E.tif]

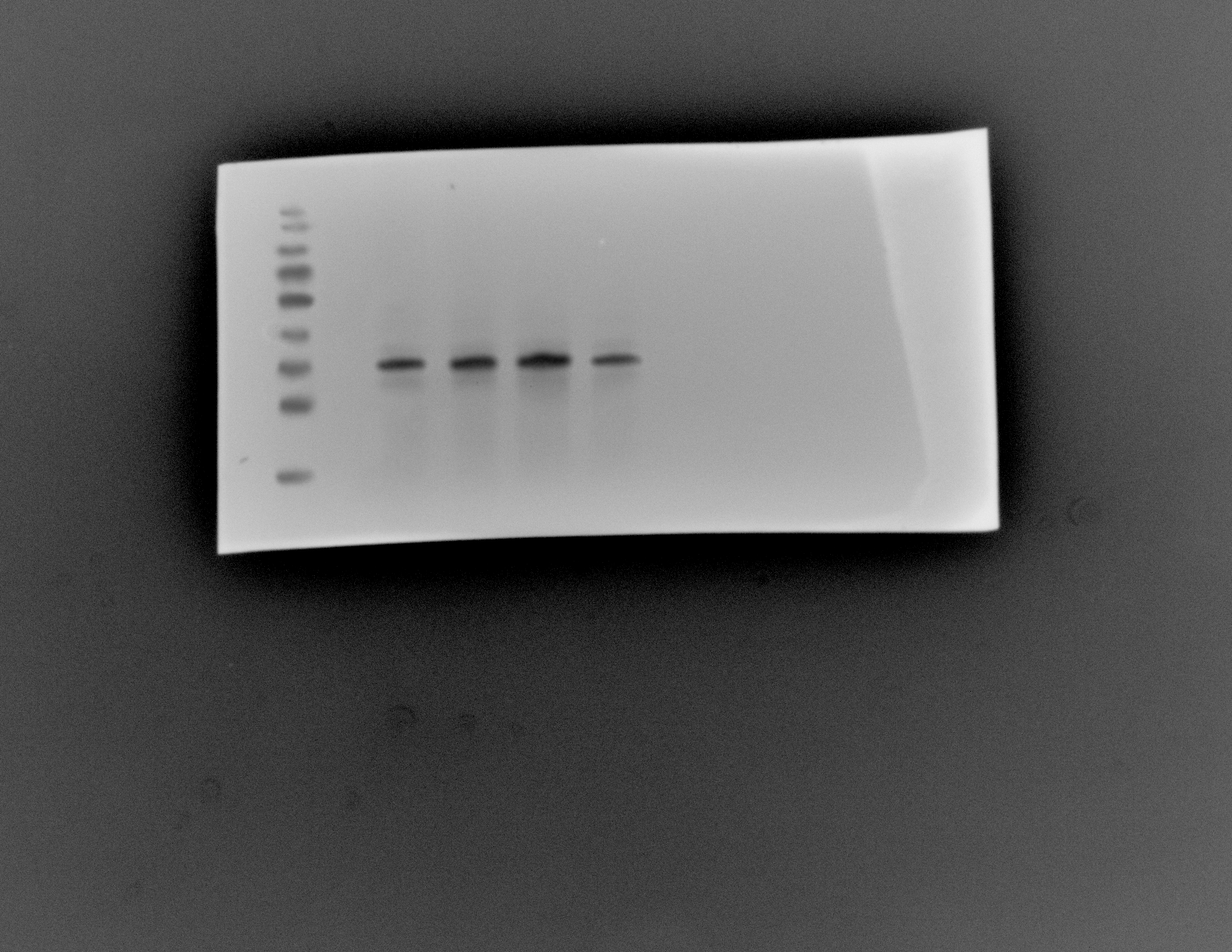

Supplement: S1 File — (ZIP) [file pone.0307667.s003.zip › figure 5- WB/Figure 5D_BCL-2_NRK-52E.tif]

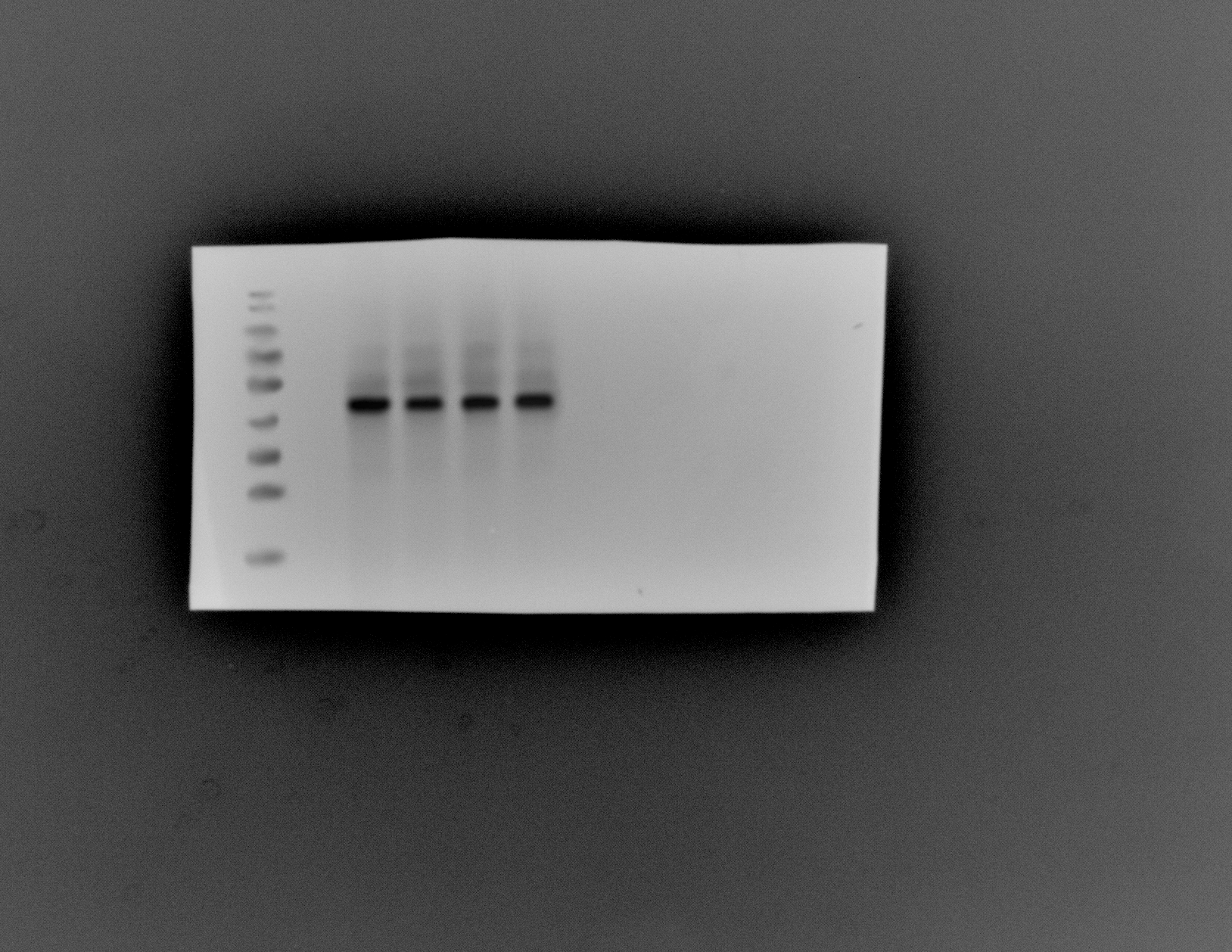

Supplement: S1 File — (ZIP) [file pone.0307667.s003.zip › figure 5- WB/Figure 5D_β-actin_NRK-52E.tif]

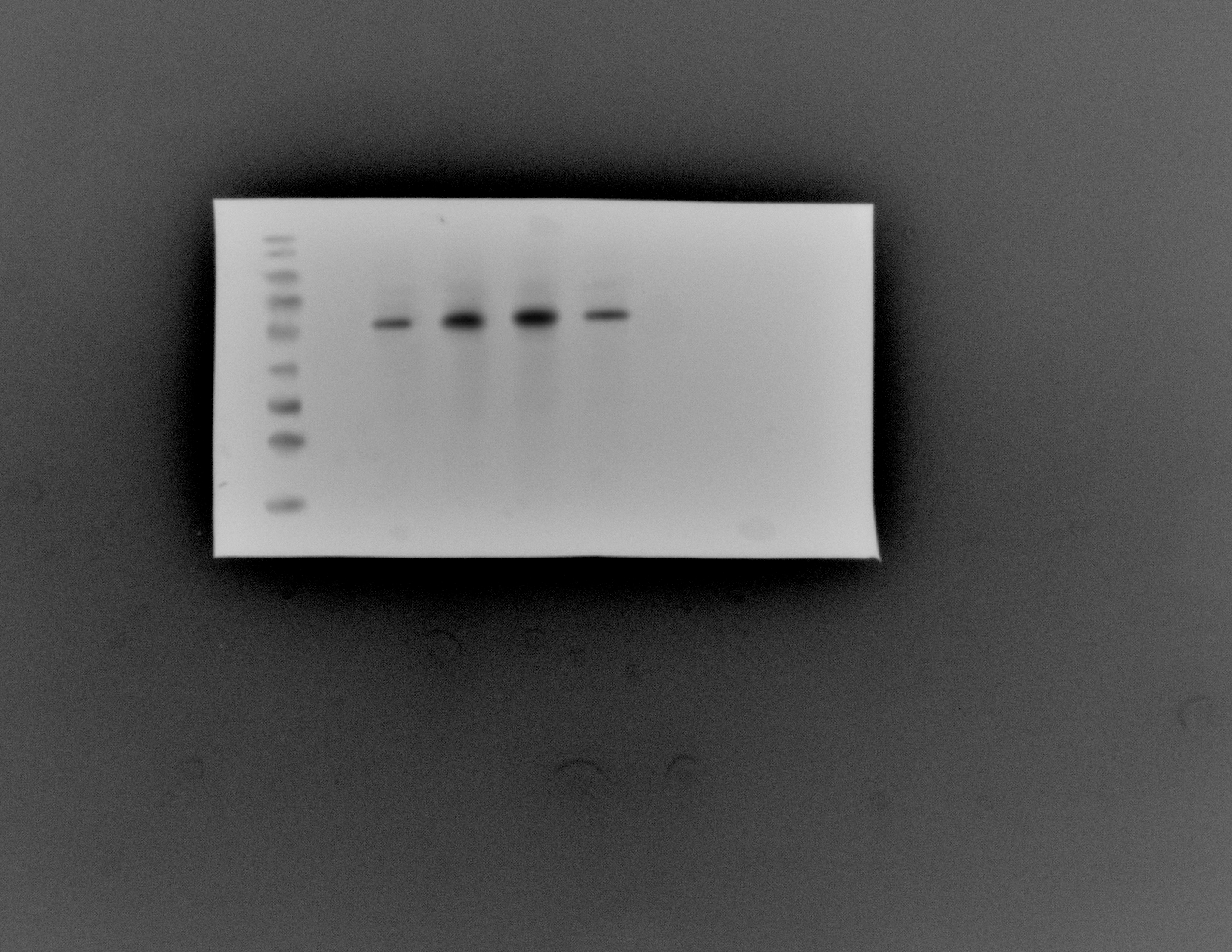

Supplement: S1 File — (ZIP) [file pone.0307667.s003.zip › figure 5- WB/Figure 5E_c-myc_NRK-52E.tif]

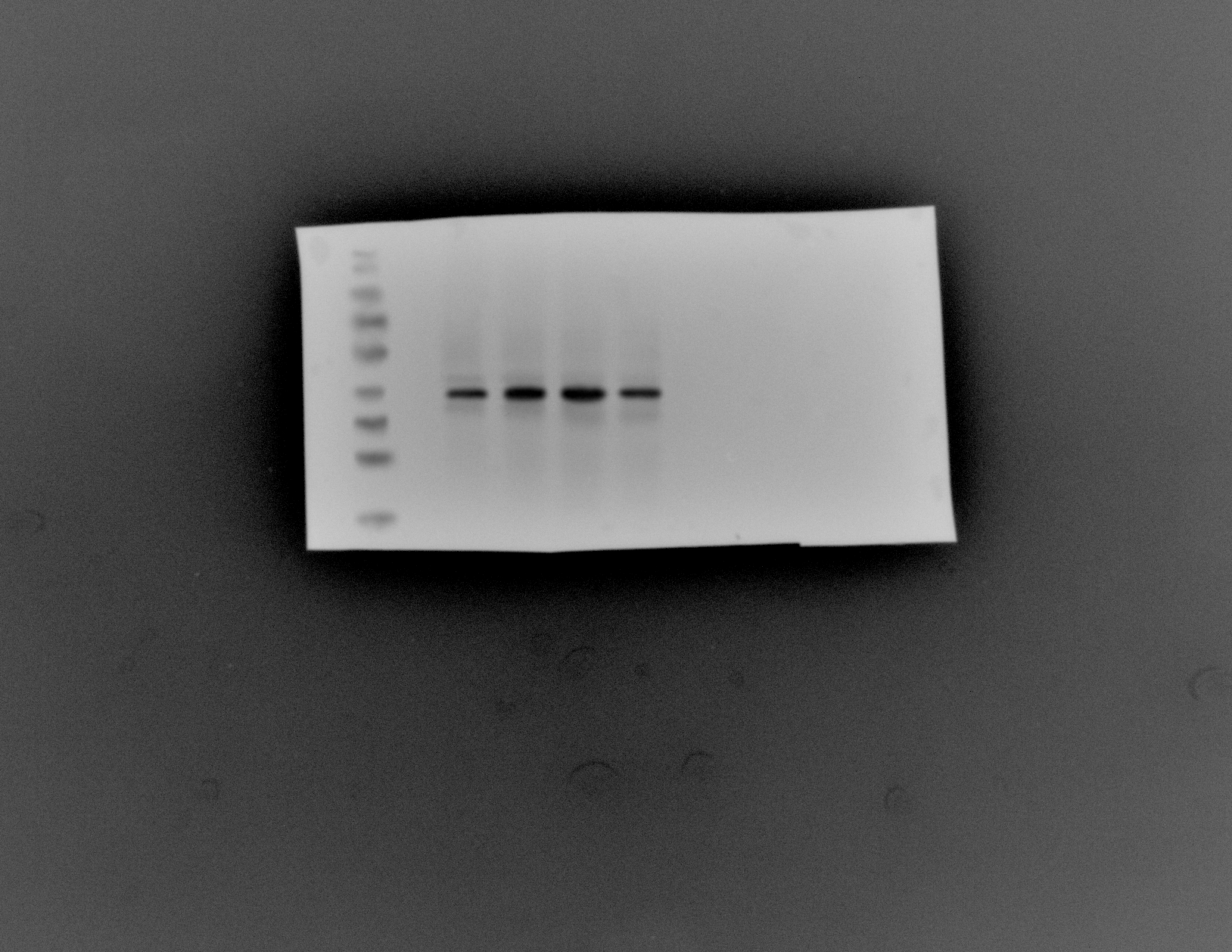

Supplement: S1 File — (ZIP) [file pone.0307667.s003.zip › figure 5- WB/Figure 5E_cyclin D1_NRK-52E.tif]

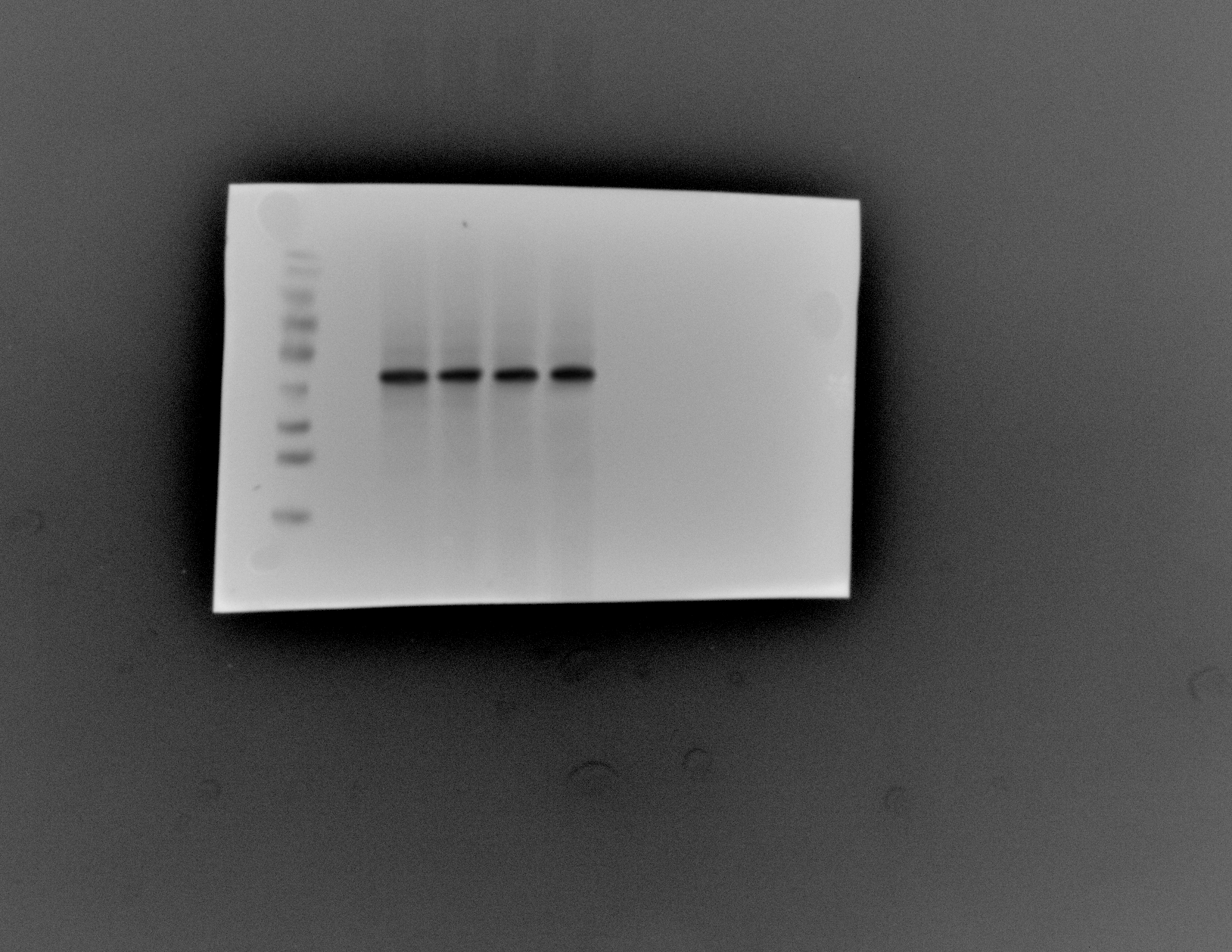

Supplement: S1 File — (ZIP) [file pone.0307667.s003.zip › figure 5- WB/Figure 5E_β-actin_NRK-52E.tif]

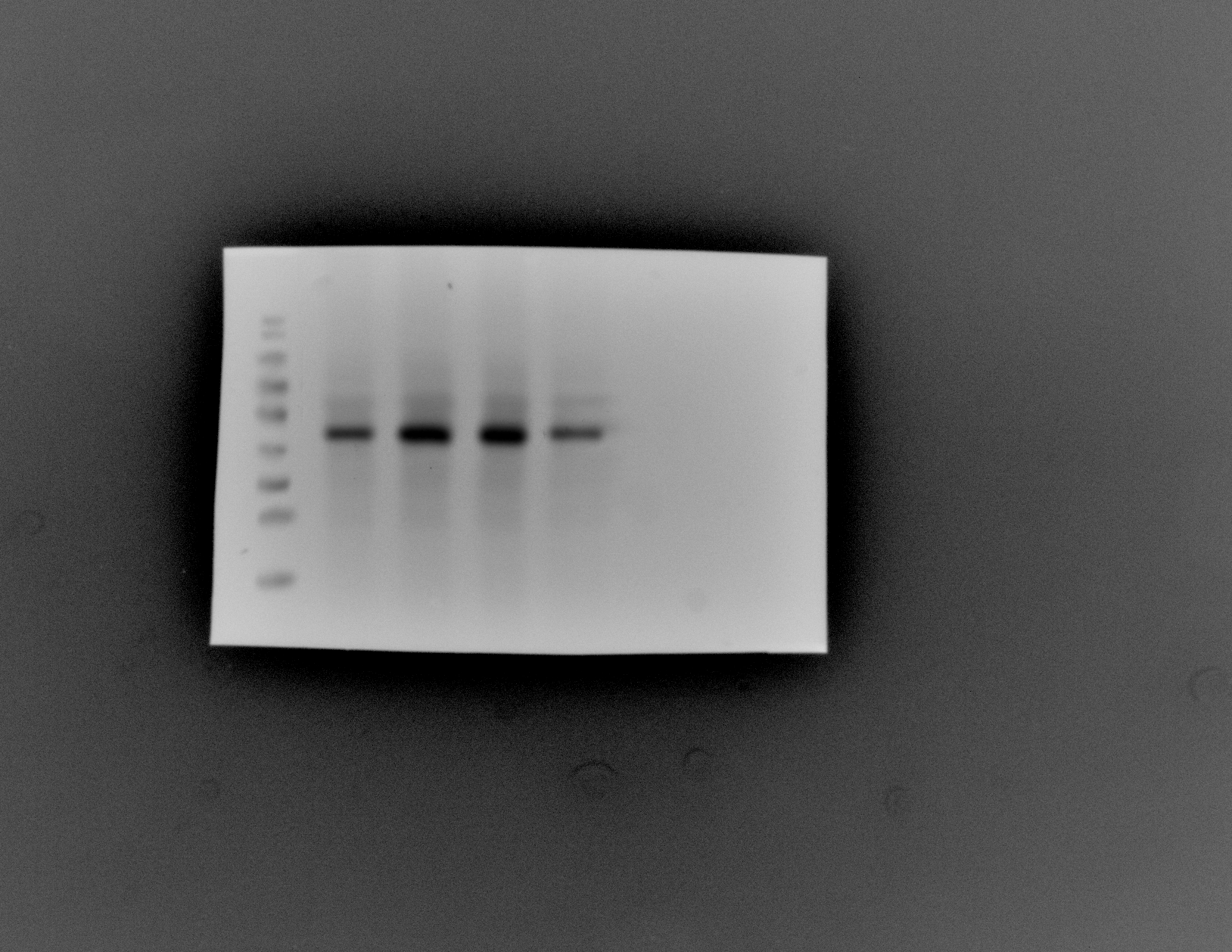

Supplement: S1 File — (ZIP) [file pone.0307667.s003.zip › figure 5- WB/Figure 5E_β-catein_NRK-52E.tif]

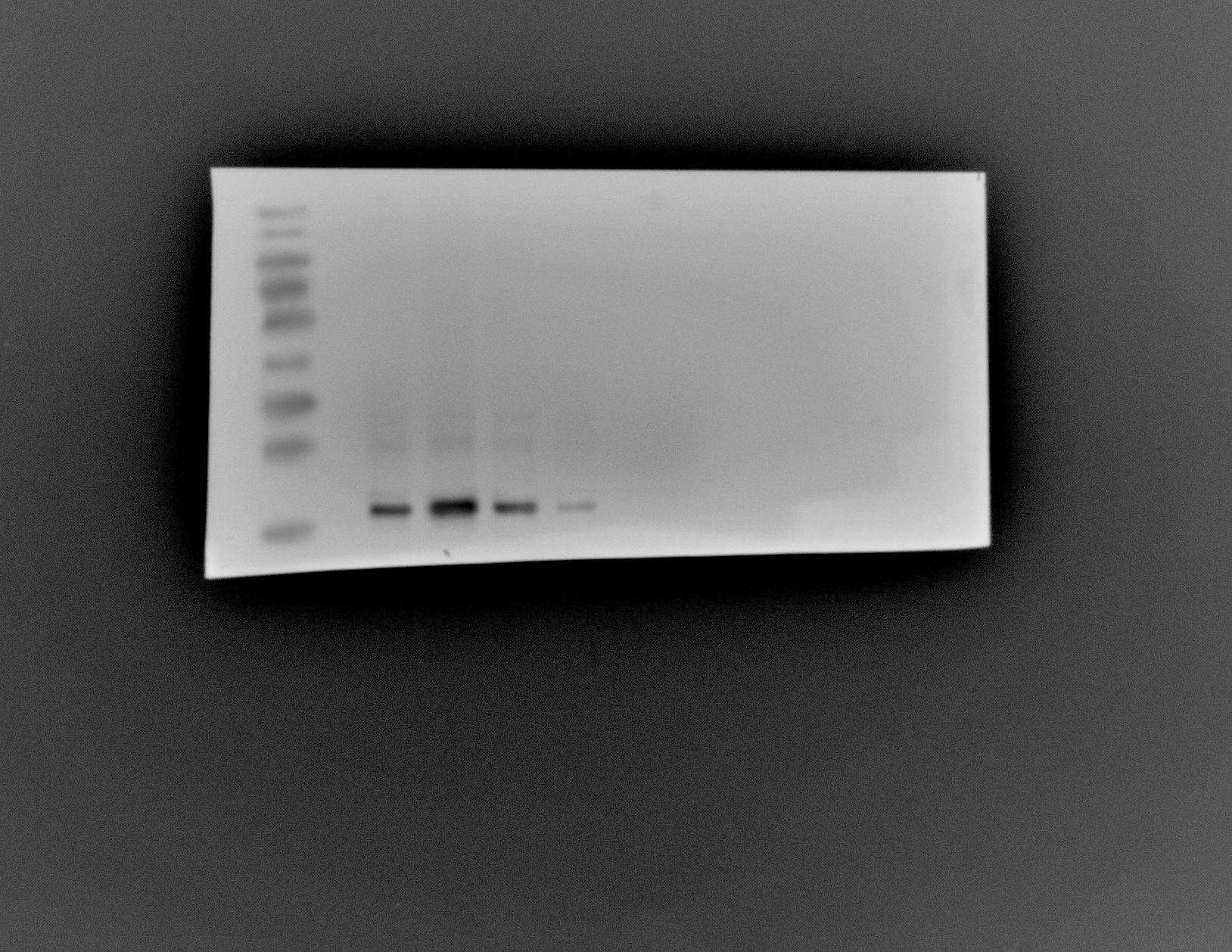

Supplement: S2 File — (ZIP) [file pone.0307667.s004.zip › figure 4- WB/Figure 4C_COX7A2L_NRK-52E-12KDa.tif]

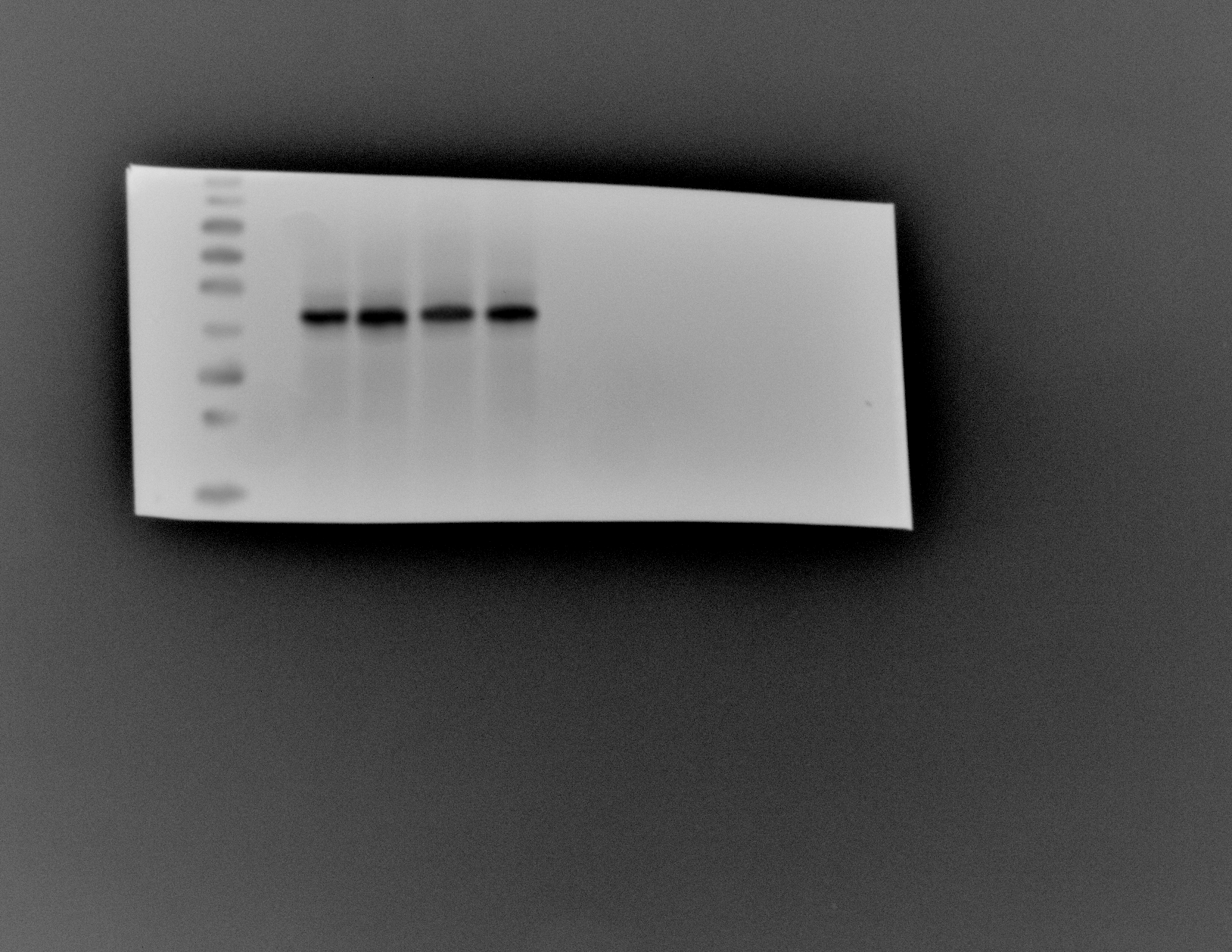

Supplement: S2 File — (ZIP) [file pone.0307667.s004.zip › figure 4- WB/Figure 4C_β-actin_NRK-52E.tif]

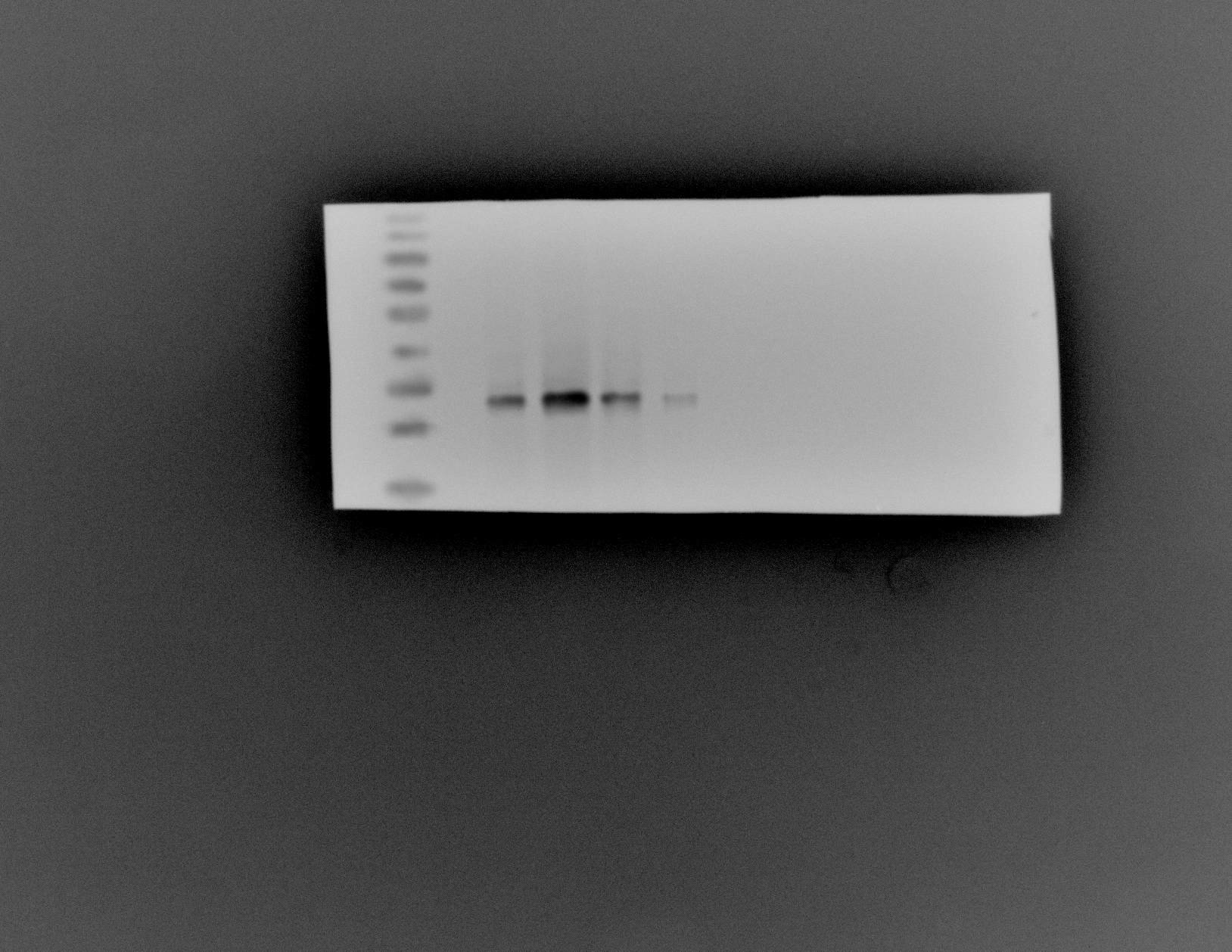

Supplement: S2 File — (ZIP) [file pone.0307667.s004.zip › figure 4- WB/Figure 4E_BAX_NRK-52E.tif]

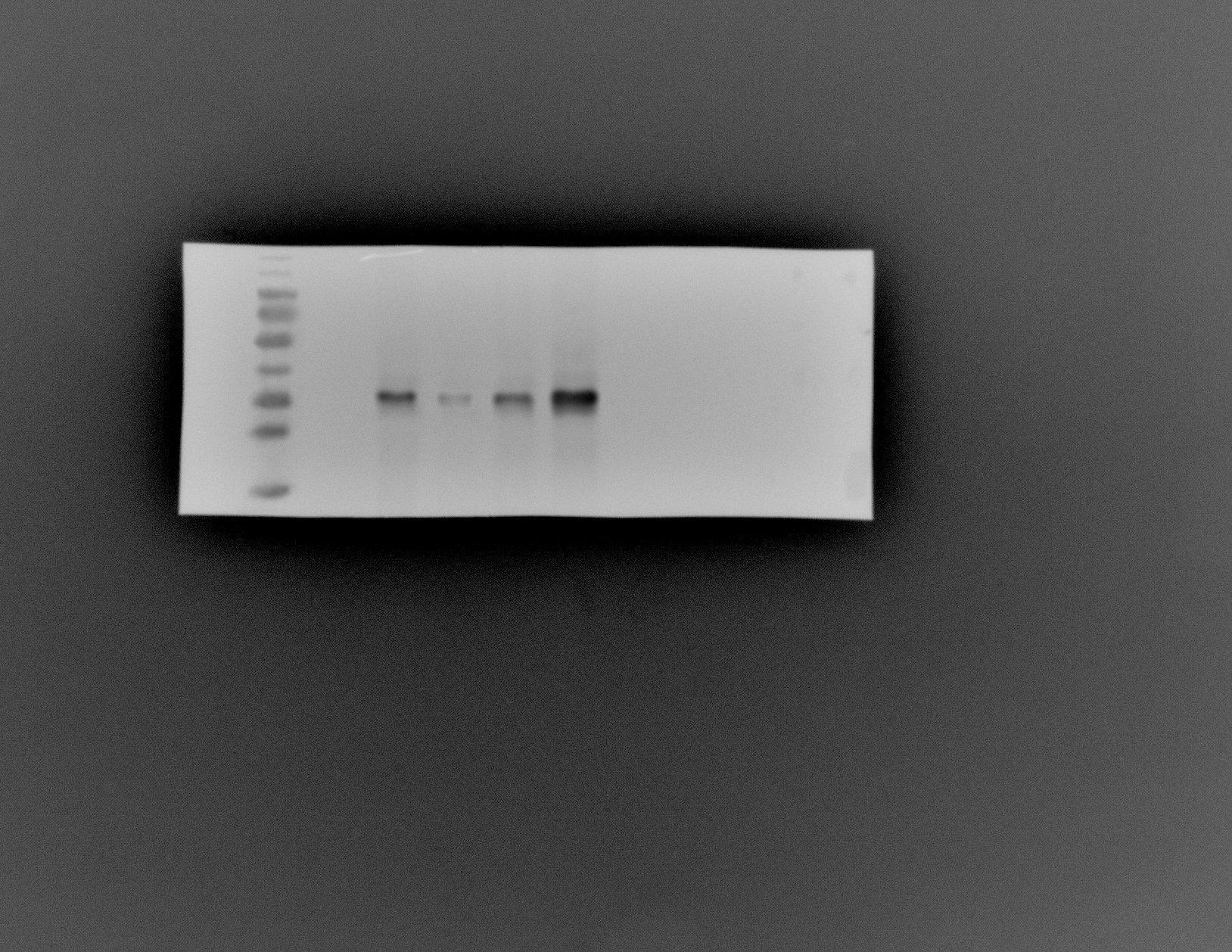

Supplement: S2 File — (ZIP) [file pone.0307667.s004.zip › figure 4- WB/Figure 4E_BCL-2_NRK-52E.tif]

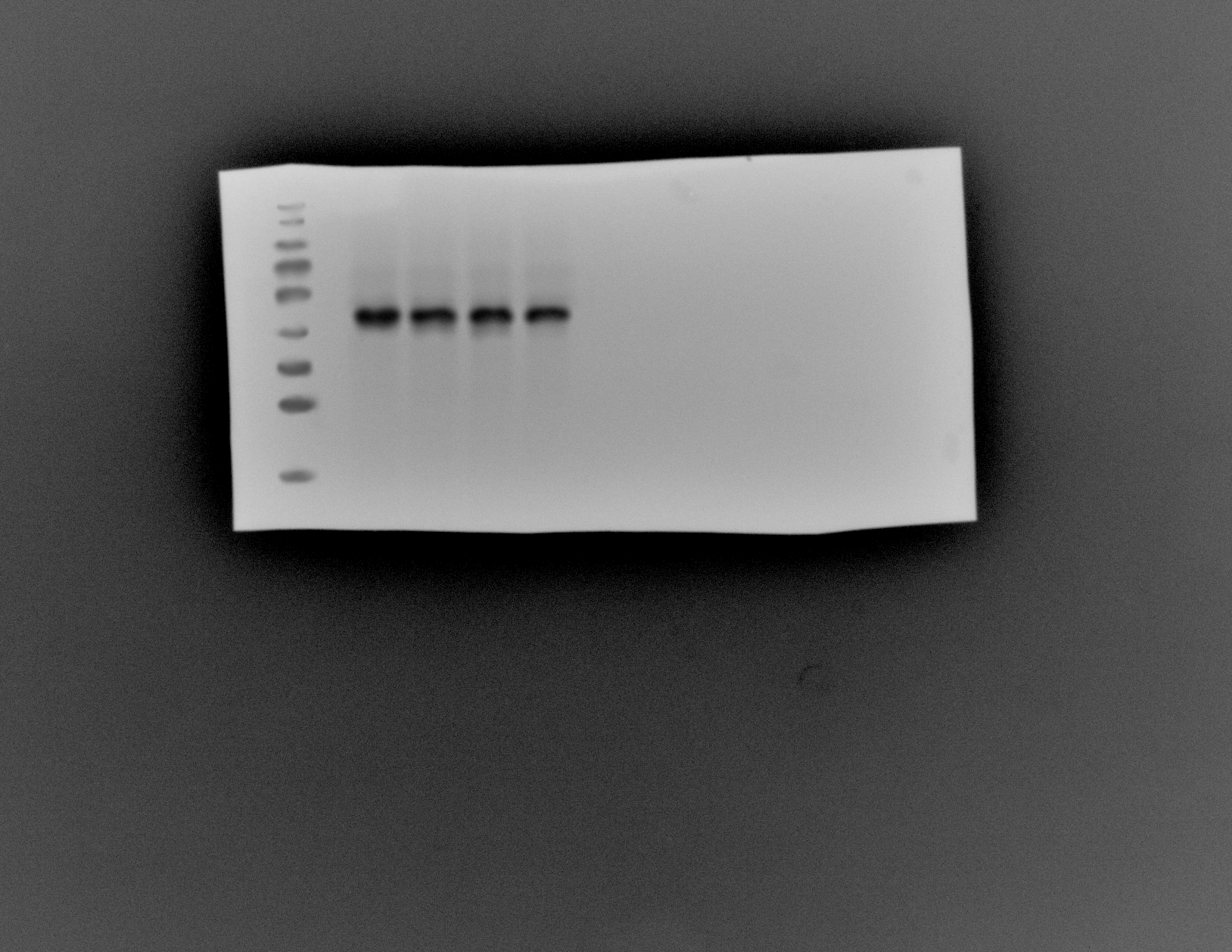

Supplement: S2 File — (ZIP) [file pone.0307667.s004.zip › figure 4- WB/Figure 4E_β-actin_NRK-52E.tif]
